# DPP4 inhibition impairs senohemostasis to improve plaque stability in atherosclerotic mice

**DOI:** 10.1172/JCI165933

**Published:** 2023-06-15

**Authors:** Allison B. Herman, Dimitrios Tsitsipatis, Carlos Anerillas, Krystyna Mazan-Mamczarz, Angelica E. Carr, Jordan M. Gregg, Mingyi Wang, Jing Zhang, Marc Michel, Charnae’ A. Henry-Smith, Sophia C. Harris, Rachel Munk, Jennifer L. Martindale, Yulan Piao, Jinshui Fan, Julie A. Mattison, Supriyo De, Kotb Abdelmohsen, Robert W. Maul, Toshiko Tanaka, Ann Zenobia Moore, Megan E. DeMouth, Simone Sidoli, Luigi Ferrucci, Yie Liu, Rafael de Cabo, Edward G. Lakatta, Myriam Gorospe

**Affiliations:** 1 Laboratory of Genetics and Genomics,; 2 Laboratory of Cardiovascular Sciences,; 3 Translational Gerontology Branch, and; 4 Laboratory of Molecular Biology and Immunology, National Institute on Aging (NIA) Intramural Research Program (IRP), NIH, Baltimore, Maryland, USA.; 5 Department of Microbiology and Immunology and; 6 Department of Biochemistry, Albert Einstein College of Medicine, Bronx, New York, USA.

**Keywords:** Aging, Vascular Biology, Atherosclerosis, Cellular senescence

## Abstract

Senescent vascular smooth muscle cells (VSMCs) accumulate in the vasculature with age and tissue damage and secrete factors that promote atherosclerotic plaque vulnerability and disease. Here, we report increased levels and activity of dipeptidyl peptidase 4 (DPP4), a serine protease, in senescent VSMCs. Analysis of the conditioned media from senescent VSMCs revealed a unique senescence-associated secretory phenotype (SASP) signature comprising many complement and coagulation factors; silencing or inhibiting DPP4 reduced these factors and increased cell death. Serum samples from persons with high risk for cardiovascular disease contained high levels of DPP4-regulated complement and coagulation factors. Importantly, DPP4 inhibition reduced senescent cell burden and coagulation and improved plaque stability, while single-cell resolution of senescent VSMCs reflected the senomorphic and senolytic effects of DPP4 inhibition in murine atherosclerosis. We propose that DPP4-regulated factors could be exploited therapeutically to reduce senescent cell function, reverse senohemostasis, and improve vascular disease.

## Graphical abstract

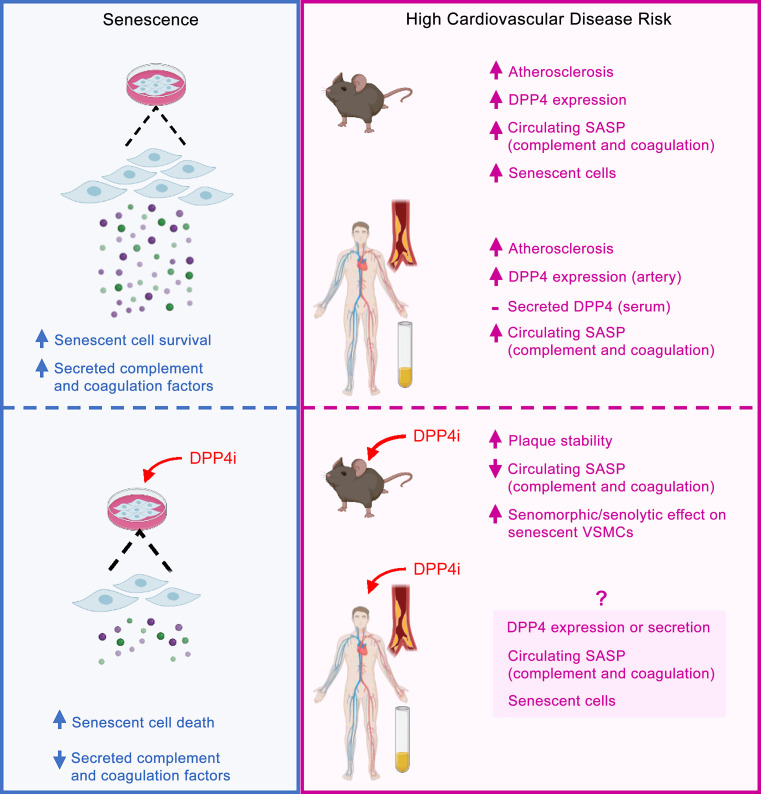

## Introduction

Atherosclerosis, the buildup of plaque and hardening of the arteries over time, is a widespread and debilitating disease in older populations. Many risk factors contribute to the development of atherosclerosis, including unhealthy diets, smoking, genetics, diabetes, aging, and obesity. Among them, aging is one of the most important factors, increasing the prevalence, incidence, and mortality associated with atherosclerosis (1, 2). The most serious complication and leading cause of death associated with atherosclerosis is the thrombotic occlusion of the arteries following erosion or rupture of an atherosclerotic plaque. The cap of the plaque consists of a layer of vascular smooth muscle cells (VSMCs) that have dedifferentiated into synthetic, noncontractile cells that typically proliferate and migrate, but also a small population of senescent, inflammatory VSMCs (3).

Previous research has shown that cellular senescence is associated with the development and progression of atherosclerosis (4). Cellular senescence, a hallmark phenotype of cells in aging tissues, is characterized by increased production of cyclin-dependent kinase inhibitors and tumor suppressors such as p16 (CDKN2A), p21 (CDKN1A), and p53 (TP53) as well as many factors of the senescence-associated secretory phenotype (SASP), including cytokines (e.g., IL-6, IL-8), chemokines (e.g., CCL2), adhesion molecules (e.g., ICAM1), matrix remodeling enzymes (e.g., MMPs), and angiogenic factors (e.g., VEGF). Senescent cells also display a senescence-associated β-galactosidase (SA–β-gal) activity (5, 6). Although senescent cells possess the ability to be both beneficial and harmful depending on the biological situation, there is little doubt that the accrual of senescent cells in older human tissues is detrimental and contributes to pathologies associated with aging (7–9). As a result of their widespread detrimental effects in many diseases, interest is escalating in developing therapies directed against senescent cells (senotherapies) and in discovering senescence-associated cell-surface proteins. Importantly, VSMCs cultured from atherosclerotic plaques display a myriad of these senescence traits; thus, further research on senescent VSMCs in atherosclerosis is crucial to our understanding of the progression and treatment of this age-associated disease (10).

The development of age-related multifactorial diseases such as atherosclerosis is associated with persistent systemic inflammation. However, the dynamic interplay among aging, inflammation, and VSMC senescence is not well understood. A recent study found that dipeptidyl peptidase 4 (DPP4) is highly expressed on the surface of senescent cells (11). While the effect of DPP4 on VSMCs is not characterized, DPP4 inhibitors, known as *gliptins*, are clinically used to treat diabetes (12). In animal models, gliptins have the ability to reduce atherosclerosis and inflammation independently of the canonical role of DPP4 in glucose metabolism (13–17). Thus, we set out to investigate the ability of DPP4 inhibitors to reduce the burden of senescent VSMCs in the progression of vascular disease.

We found that DPP4 was elevated in and on the surface of primary coronary artery human VSMCs (hVSMCs) rendered senescent by extended culture in the presence of the hypoxia mimic cobalt chloride (CoCl_2_) or the DNA-damaging agent doxorubicin (Doxo) as well as in human and mouse atherosclerotic plaques. Interestingly, silencing DPP4 or treatment with the DPP4 inhibitor vildagliptin (DPP4i) moderately reduced senescent hVSMC viability and increased apoptosis. Proteomic analysis of media from senescent hVSMCs treated with DPP4i revealed a reduction in many complement and coagulation factors, suggesting that hVSMCs possess a SASP comprising hemostatic proteins and that production of these factors is mitigated by DPP4 inhibition. Importantly, in atherosclerotic mice treated with DPP4i, we found reduced DPP4 activity, blood-clotting time, and expression of SASP factors, including coagulation factors, while plaque stability improved. In this mouse model, single-cell analysis of the aorta revealed that atherosclerosis increased the number of vascular cells expressing DPP4 on the surface; these senescent VSMCs displayed heterogenous, clustered responses to DPP4i, suppressing coagulation-related gene expression in one cell cluster and viability in another cell cluster. We propose that DPP4 promotes local cell survival of senescent vascular cells and increases plaque instability while it also stimulates secretion of complement and coagulation factors to elicit a systemic program we termed *senohemostasis*.

## Results

### Senescent hVSMCs have increased DPP4 expression levels and activity.

**Figure 1:**
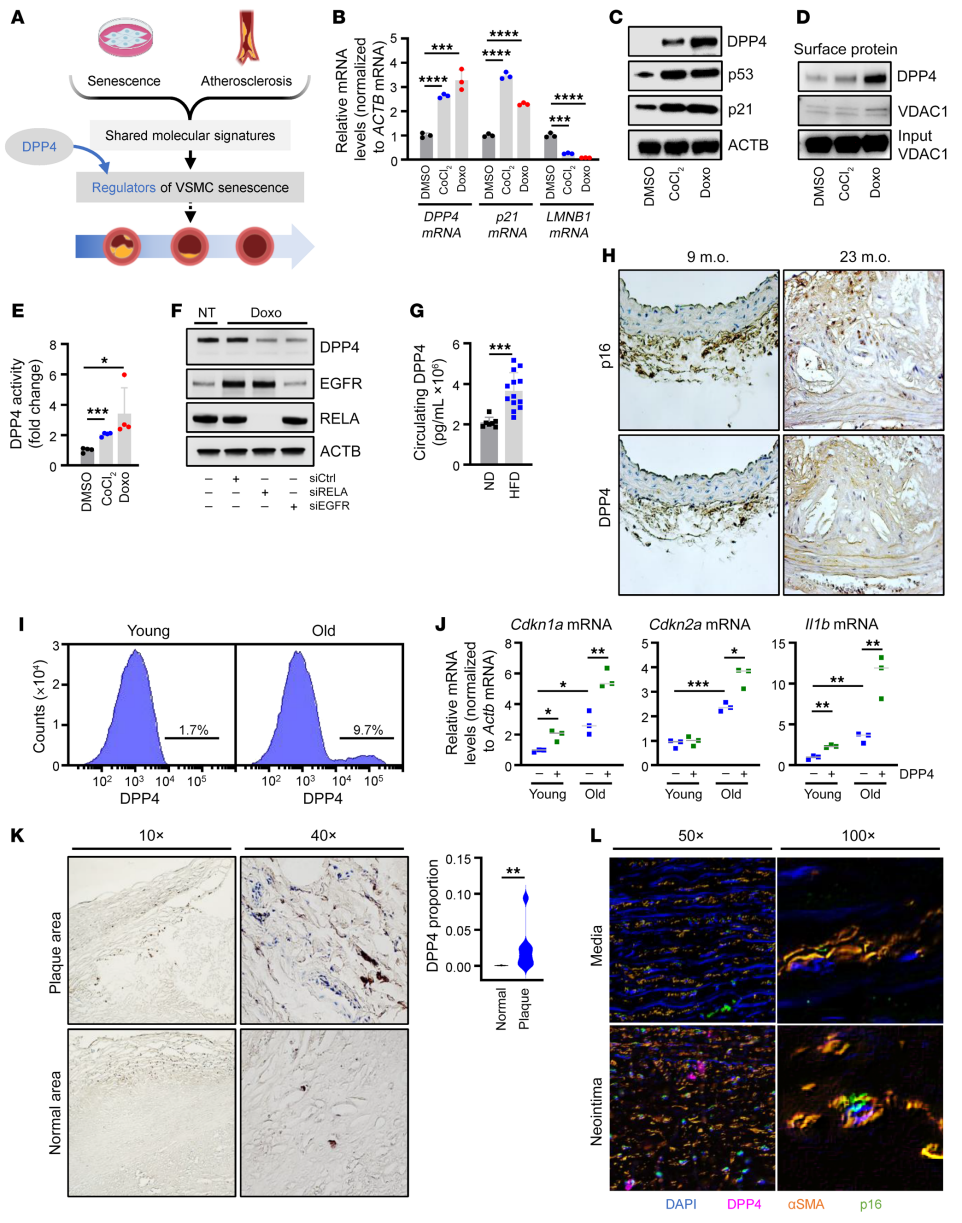
DPP4 is elevated and more active in senescent hVSMCs and mouse, monkey, and human atherosclerosis. (**A**) Schematic highlighting DPP4 function at the intersection of the senescence and atherosclerosis programs. (**B**) RT-qPCR analysis of the levels of *DPP4*, *p21*, and *LMNB1* (*Lamin B1*) mRNAs in hVSMCs treated with DMSO, CoCl_2_ (200 nM), or Doxo (125 nM) for 7 days, normalized to *ACTB* (β-actin) mRNA levels. (**C**) hVSMCs were treated as in **B**, and the levels of proteins DPP4, p53, p21, and loading control ACTB were assessed by Western blot analysis. (**D**) Cell-surface proteins were biotinylated and pulled down in hVSMCs following treatment with DMSO, CoCl_2_, or Doxo for 7 days. Protein levels were assessed by Western blot analysis for cell-surface proteins DPP4 and VDAC1; VDAC1 input was included as a loading control. (**E**) DPP4 activity was measured in hVSMCs treated with DMSO, CoCl_2_, or Doxo for 7 days. (**F**) Western blot analysis of EGFR and RELA silencing in no treatment (NT) or Doxo-treated hVSMCs. (**G**) *Ldlr^–/–^* mice were fed ND or HFD for 16 weeks, and circulating DPP4 levels were measured in serum. (**H**) Aortas from 9 or 23 m.o. *ApoE^–/–^* mice were analyzed for p16 and DPP4. Original magnification, ×100. (**I**) Flow cytometry analysis of DPP4 on the surface of aortic VSMCs from young (*n* = 10) and old (*n* = 10) C57BL/6J mice. (**J**) RT-qPCR analysis of *Cdkn1a*, *Cdkn2a*, and *Il1b* mRNAs normalized to *Actb* mRNA from DPP4-positive or negative mouse VSMCs from young (*n* = 9, pooled 3 per data point) and old mice (*n* = 9, pooled 3 per data point) (Supplemental Figure 2A). (**K**) DPP4 in human aortic tissue (*n* = 5) was analyzed by IHC. Left: top row demonstrates DPP4 expression in plaque area; bottom row represents normal or nonplaque area of the same tissue. Right: analysis of the proportion of DPP4 in plaque and normal tissue in the total tissue area. Original magnification, ×10 (left panels); ×40 (right panels). (**L**) Immunofluorescent analysis of human atherosclerotic aortic media and neointima to colocalize DAPI (blue), DPP4 (purple), αSMA (orange), and p16 (green) signals. Original magnification, ×50 (left panels); ×100 (right panels). Data are represented as mean ± SD from *n* = 3 or otherwise indicated biological replicates. Significance was established using Student’s *t* test with Bonferroni’s correction. *P ≤ 0.05; **P ≤ 0.01; ***P ≤ 0.001; ****P ≤ 0.0001.

We investigated the protein DPP4 (also known as CD26), previously found on the surface of senescent fibroblasts (11) (Figure 1A), to assess its role in hVSMC senescence. DPP4 is an exopeptidase that selectively cleaves N-terminal dipeptides from a variety of substrates and is best known for cleaving and inactivating glucagon-like peptide-1 (GLP-1) to disrupt glucose homeostasis associated with type 2 diabetes (18, 19). To begin to assess the connection between senescence and DPP4 in hVSMCs, we induced hVSMC senescence using 2 different paradigms: treatment for 7 days with CoCl_2_, a hypoxia mimetic, or with Doxo, a chemotherapeutic drug that induces DNA damage (20, 21). Cells were then collected, and the levels of *DPP4* mRNA and the senescence markers *p21* and *LMNB1* mRNAs were measured in proliferating and senescent VSMCs by reverse transcription followed by quantitative PCR analysis (RT-qPCR) (Figure 1B). We also confirmed that the levels of senescence proteins DPP4, p21, and p53 increased under the same conditions (Figure 1C). DPP4 was similarly elevated in human diploid WI-38 fibroblasts following senescence-inducing treatments such as etoposide (ETO) or ionizing radiation (IR) as well as by oxidative stress–induced (OS-induced) senescence and in human renal mixed epithelial cells (HRECs) rendered senescent by treatment with ETO (Supplemental Figure 1, A and B; supplemental material available online with this article; https://doi.org/10.1172/JCI165933DS1), supporting the view that DPP4 upregulation may be broadly conserved among different senescent cell types.

We next asked whether DPP4 protein is expressed on the cell surface of senescent hVSMCs, as previously discovered on fibroblasts. Using a protocol that entailed biotinylating cell-surface proteins in live cells before harvesting, we confirmed the expression of DPP4 on the surface of senescent hVSMCs by Western blot analysis (Figure 1D). By flow cytometry analysis using an antibody directed at DPP4, we further verified that the levels of DPP4 on the cell surface increased in hVSMCs rendered senescent by exposure to either CoCl_2_ or Doxo (Supplemental Figure 1C). In addition to its presence on the cell surface, we also investigated whether the proteolytic activity of DPP4 was altered during senescence. Using a DPP4 activity assay (see Methods), we observed higher DPP4 peptidase activity in hVSMCs treated with either CoCl_2_ or Doxo (Figure 1E). Together, these data indicate that whole-cell DPP4, cell-surface DPP4, and DPP4 enzymatic activity increased in senescent hVSMCs.

To identify the mechanisms by which DPP4 expression levels increased in senescent hVSMCs, we focused on transcription, based on earlier studies of DPP4 production (11, 22). Consensus sequences for transcription factors (TFs) NF-κB, SP1, EGFR, and AP-1 were previously found in the *DPP4* promoter (23). To determine whether any of these TFs might regulate DPP4 levels in senescent hVSMCs, we transfected specific siRNAs designed to silence RELA (a member of the NF-κB family), SP1, EGFR, or FOS (a member of the AP-1 family), along with a control siRNA (siCtrl); 72 hours later, we triggered senescence by treatment with Doxo. After an additional 72 hours, we assessed the levels of mRNAs encoding these TFs and discovered that the levels of *RELA* and *EGFR* mRNAs increased, while the levels of *SP1* and *FOS* mRNAs declined in response to Doxo-induced senescence (Supplemental Figure 1, D–G). Furthermore, depleting RELA or EGFR strongly reduced the levels of *DPP4* pre-mRNA and mature *DPP4* mRNA, suggesting that RELA and EGFR transcriptionally activate DPP4 production (Figure 1F and Supplemental Figure 1, D and E). On the other hand, silencing SP1 or FOS did not alter *DPP4* pre-mRNA levels, despite reducing the levels of mature *DPP4* mRNA, suggesting that their effects could be indirect (Supplemental Figure 1, F and G).

### DPP4 protein levels and activity increase with atherosclerosis in mouse, monkey, and human.

Given that DPP4 levels and activity increase with hVSMC senescence, we asked whether DPP4 is elevated in atherosclerosis, a disease in which senescent VSMCs have been implicated (24). DPP4 on the plasma membrane can be cleaved, releasing soluble DPP4 into circulation; therefore, we determined whether the serum levels of DPP4 changed in mouse models of atherosclerosis. *Ldlr^–/–^* mice fed a high-fat diet (HFD) for 16 weeks demonstrated significantly higher levels of circulating DPP4 protein and DPP4 activity than mice fed a normal diet (ND) (Figure 1G and Supplemental Figure 1H). In older (23 months old [m.o.]) *ApoE^–/–^* mice, which are genetically modified mice often used to study atherosclerosis, the expression levels of both the senescent markers p16 and DPP4 increased in the plaque area compared with those of control mice (Figure 1H and Supplemental Figure 1I). To further assess possible links between DPP4 and senescence in murine VSMCs, we compared senescence-associated gene expression patterns between the DPP4^+^ and DPP4^–^ VSMC populations from young (3 m.o.) and old (30 m.o.) C57BL/6 mice. These results revealed that old mice, which harbor a greater number of senescent cells (25), have a higher percentage of DPP4^+^ VSMCs compared with young mice (Figure 1I). In addition, when compared with DPP4^–^ VSMCs, DPP4^+^ VSMCs from old mice (and in some cases young mice) expressed higher levels of senescence-associated markers *p16*, *p21*, *Gdf15*, and *Il1b* mRNAs, which increase with senescence, while they expressed lower levels of *Lmnb1* mRNA, which declines with senescence (26) (Figure 1J and Supplemental Figure 2A). We next examined aortic tissue collected from young and old rhesus monkeys from the NIA rhesus colony participating in an ongoing study that were fed HFD or ND for 2 years. Here too, higher DPP4 levels correlated with increasing age and with HFD (Supplemental Figure 2B); consistent with these findings, circulating DPP4 levels and activity in the serum from these monkeys increased most significantly in old monkeys fed HFD (Supplemental Figure 2C).

We then evaluated human atherosclerotic tissue and discovered that DPP4 protein was elevated in atherosclerotic plaques, specifically in and around the plaque cap, suggesting that immune cells and proinflammatory hVSMCs express high levels of DPP4 (Figure 1K and Supplemental Figure 2D). Immunofluorescent staining of older human atherosclerotic tissue confirmed these findings, as demonstrated by the colocalization of p16 and DPP4 in VSMCs (αSMA-positive), specifically those within the neointima region surrounding the plaque (Figure 1L and Supplemental Figure 2E). In addition, we surveyed human serum samples from the Baltimore Longitudinal Study of Aging (BLSA) (27) from people aged 50 to 65 with low and high Framingham risk scores (FRS) (28), which estimate the risk of cardiovascular disease, for circulating levels of DPP4. The levels of DPP4 were unchanged regardless of FRS, supporting the view that DPP4 itself is not an ideal circulating biomarker and may instead exert its function locally upon nearby cells and tissues (Supplemental Figure 2F). Whether circulating DPP4 is more active than its membrane-bound counterpart remains unclear, but DPP4 is increased locally in atherosclerosis with cell senescence and in vascular disease (Figure 1, K and L).

### Loss or inhibition of DPP4 induces senescent cell death.

**Figure 2:**
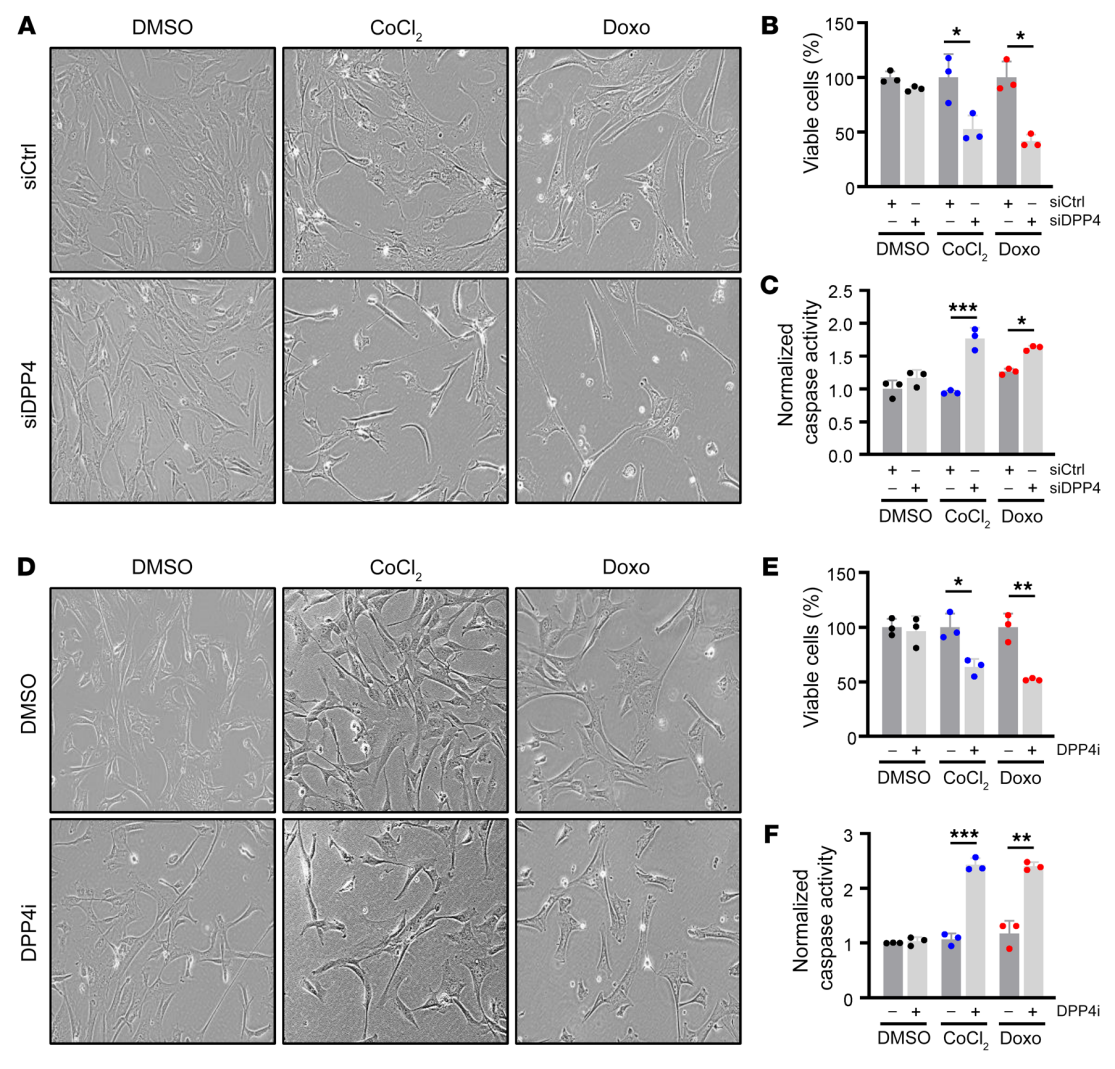
Loss or inhibition of DPP4 moderately promotes death of senescent hVSMCs. (**A**) Micrographs depicting bright-field images of hVSMCs transfected with siCtrl or siDPP4 and treated with DMSO, CoCl_2_, or Doxo for 7 days. Original magnification, ×20. (**B**) Percentage of viable cells from conditions described in **A**. (**C**) Normalized caspase-3 and -7 activity levels for cells and conditions described in **A**. (**D**–**F**) hVSMCs treated simultaneously with DMSO or DPP4i and DMSO, CoCl_2_, or Doxo for 7 days were visualized (**D**) and the percentage of viable cells (**E**), and normalized caspase-3 and -7 activity levels (**F**) were calculated. Original magnification, ×20. Data are represented as mean ± SD from *n* = 3 or indicated biological replicates. Significance was established using Student’s *t* test with Bonferroni’s correction. *P ≤ 0.05; **P ≤ 0.01; ***P ≤ 0.001.

In light of the increase in DPP4 levels and activity in senescent hVSMCs and confirmed in various models of atherosclerosis, we sought to understand the role of DPP4 on senescent hVSMCs. We transfected proliferating hVSMCs with siRNAs directed against DPP4 (siDPP4) or siCtrl; 24 hours later, we treated the cells with CoCl_2_ or Doxo and assessed senescence 7 days after that. As shown (Figure 2, A and B), the loss of DPP4 increased cell death specifically in senescent cells; this result was validated by using bright-field imaging and calculating the percentage of cell viability by counting cells remaining compared with the number of cells present in the control conditions (see Methods), which collectively reflected apoptotic morphologies and reduced cell numbers. Interestingly, not all DPP4-silenced senescent cells underwent apoptosis, suggesting the effect is heterogeneous, as supported by a modest but significant increase in the activities of proapoptotic caspases 3 and 7, an assay that evaluates apoptosis (Figure 2C). Next, we considered that pharmacological inhibition of DPP4, which is clinically used to treat diabetes, is also effective at reducing atherosclerotic burden in mice (29). As shown, simultaneous treatment of hVSMCs with the DPP4i (20 μM) and with senescence inducers (CoCl_2_ or Doxo) had effects similar to that of silencing DPP4, as evidenced by reduced senescent cell viability and elevated caspase-3 and -7 activity (Figure 2, D-F). Time-course analysis of DPP4i on senescent hVSMCs corroborated these findings, showing that inhibition of DPP4 begins to induce apoptosis as senescence develops (Supplemental Figure 3).

**Figure 3:**
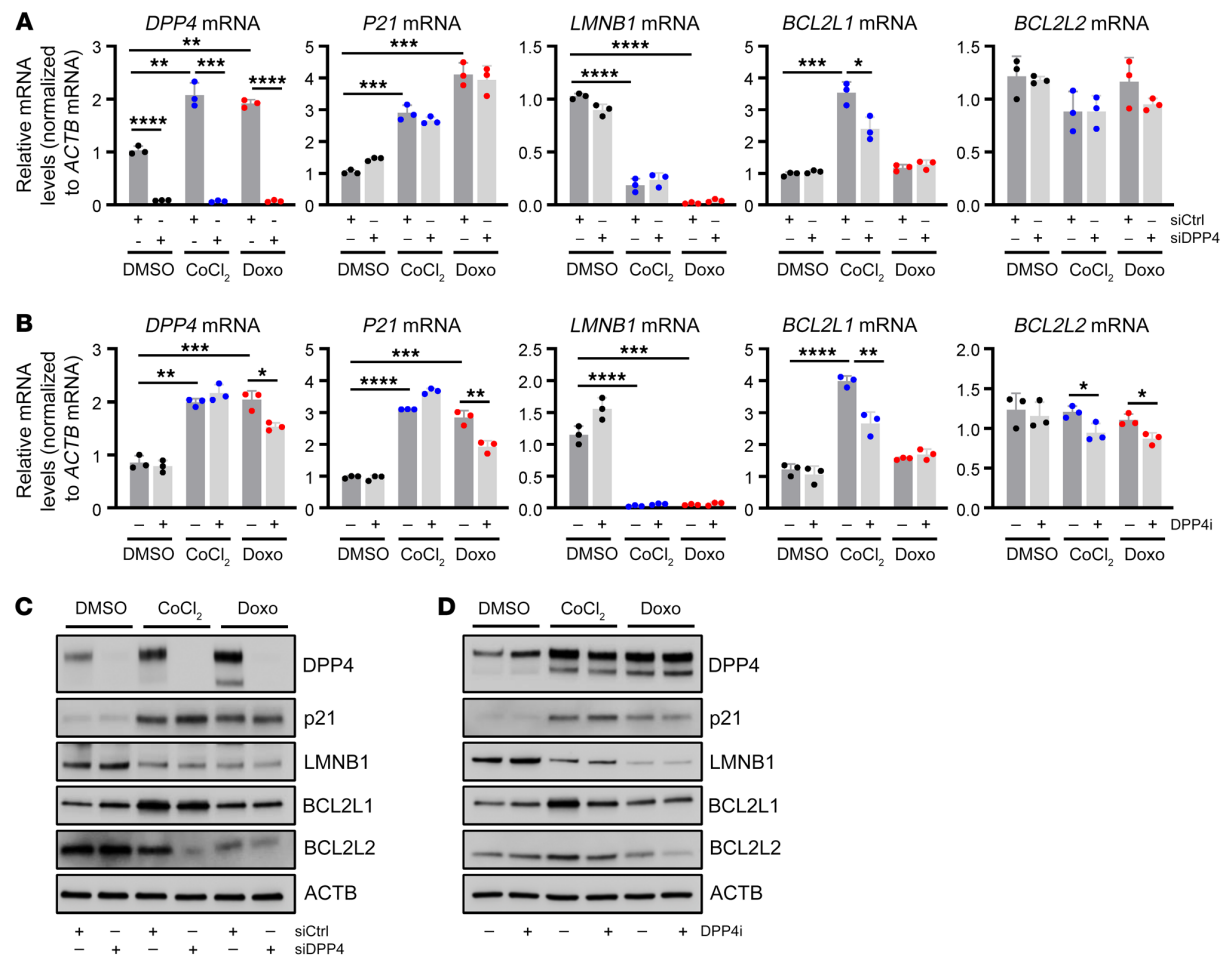
Loss or inhibition of DPP4 reduces cell-survival proteins BCL2L1 and BCL2L2. (**A**) RT-qPCR analysis of RNA from hVSMCs transfected with siCtrl or siDPP4 and treated with DMSO, CoCl_2_, or Doxo for 7 days. Graphs indicate the levels of *DPP4*, *p21*, *LMNB1*, *BCL2L1*, and *BLC2L2* mRNAs in each group, normalized to *ACTB* mRNA levels. (**B**) RT-qPCR analysis of RNA from hVSMCs transfected with siCtrl or siDPP4 and treated with DMSO, CoCl_2_, or Doxo for 7 days. The levels of *DPP4*, *p21*, *LMNB1*, *BCL2L1*, and *BLC2L2* mRNAs were normalized to *ACTB* mRNA levels. (**C**) Western blot analysis of protein levels of p21, LMNB1, BCL2L1, BCL2L2, and loading control ACTB in hVSMCs processed as described in **A**. (**D**) Western blot analysis of the levels of p21, LMNB1, BCL2L1, BCL2L2, and loading control ACTB in hVSMCs treated as described in **B**. Data are represented as mean ± SD from *n* = 3 or indicated biological replicates. Significance was established using Student’s *t* test with Bonferroni’s correction. *P ≤ 0.05; **P ≤ 0.01; ***P ≤ 0.001; ****P ≤ 0.0001.

Although transfecting siDPP4 strongly reduced *DPP4* mRNA levels, it had no detectable effect on the levels of senescence markers such as *p21* mRNA, *LMNB1* mRNA, or SA–β-gal activity relative to cell number (Figure 3A and Supplemental Figure 4A), although it reduced the levels of *BCL2L1* mRNA (Figure 3A), encoding the survival factor BCL2L1 (BCLXL) in CoCl_2_-induced senescence. DPP4i treatment was also ineffective at increasing *LMNB1* mRNA or reducing SA–β-gal activity relative to cell number, but it reduced the levels of *BCL2L2* mRNA, encoding the survival protein BCL2L2 (BCLW) in senescent hVSMCs (Figure 3B and Supplemental Figure 4B). Consistent with these findings, BCL2L2 protein levels were reduced by siDPP4 and DPP4i in senescent hVSMCs, while p21 and LMNB1 remained unchanged (Figure 3, C and D). Silencing DPP4 in senescent WI-38 fibroblasts also increased cell death, further suggesting that the promotion of survival by DPP4 may be conserved among senescent cells (Supplemental Figure 4, C and D). Together, these data indicate that DPP4 may solidify hVSMC senescence at least in part by promoting the viability of senescent cells.

### Senescent hVSMCS secrete procoagulation and complement factors that are suppressed by DPP4 inhibition.

**Figure 4:**
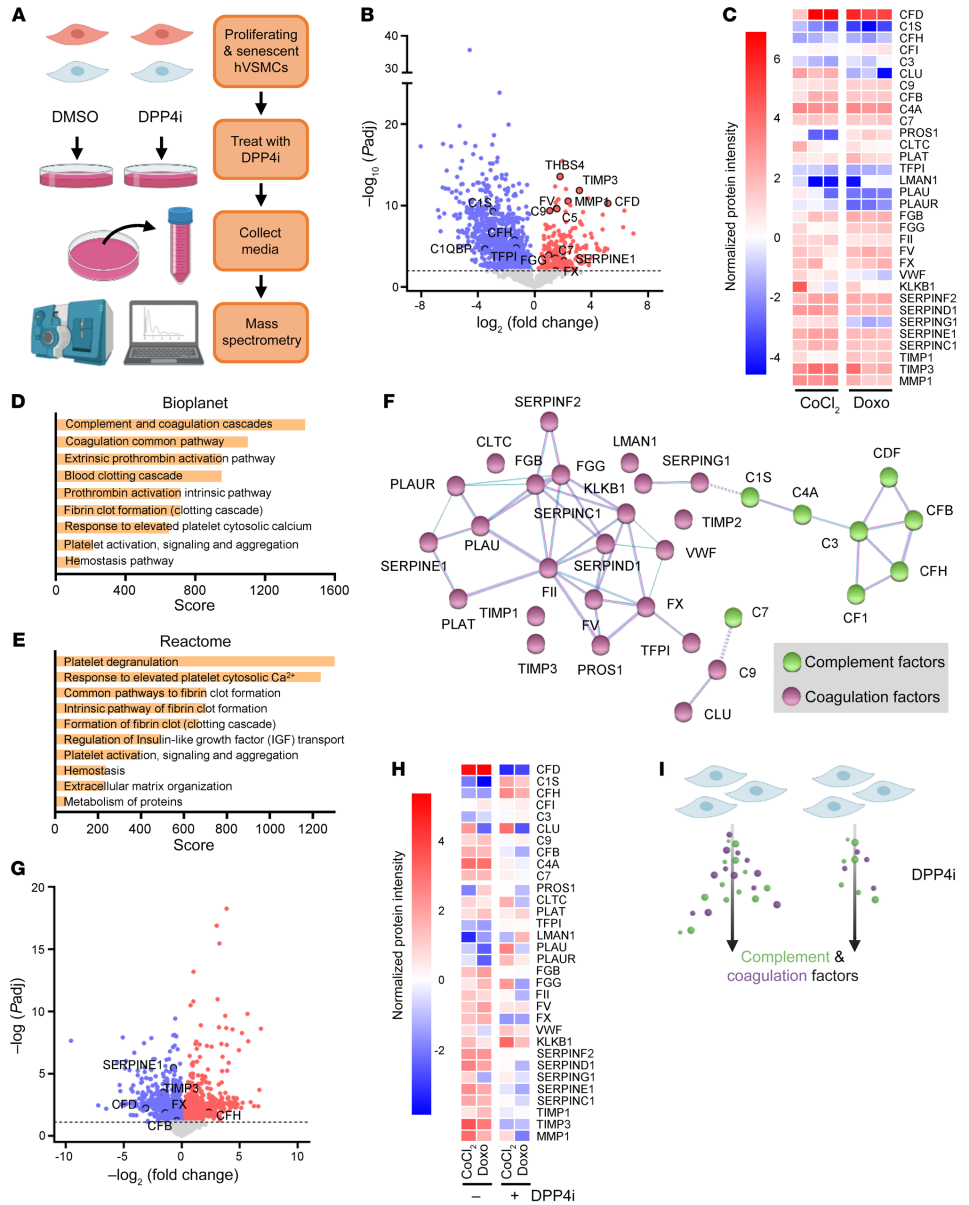
Senescent hVSMCS secrete procoagulation and complement factors that are suppressed by DPP4 inhibition. (**A**) Schematic depicting the experimental setup for mass spectrometry analysis of the media of proliferating and senescent hVSMCs treated with and without DPP4i. (**B**) Volcano plot of the proteins increased (red) and decreased (blue) in senescent hVSMCs compared with proliferating hVSMCs. Proteins in red and blue have a significance (–log_10_ adjusted *P* value [*P*adj]) ≥2. (**C**) Heatmap of the complement and coagulation proteins in each CoCl_2_ and Doxo replicate (*n* = 3) compared with the mean of the proliferating control for each protein. (**D**) Top 10 pathways enriched using BioPlanet pathway analysis ranked by combined score in senescent hVSMCs. (**E**) Reactome pathway analysis as ranked by combined score representative of the top elevated proteins in senescent hVSMCs. (**F**) STRING functional protein association network analysis to determine protein interactions among the top elevated proteins in senescent hVSMCs. (**G**) Volcano plot of proteins increased (red) and decreased (blue) after DPP4i treatment of senescent hVSMCs. Proteins in red and blue have a significance (–log_10_ *P*adj) ≥ 1. (**H**) Heatmap of complement and coagulation proteins in senescent hVSMCs (mean of CoCl_2_ and Doxo replicates, *n* = 3) compared with senescent hVSMCs treated with DPP4i (mean of CoCl_2_ and Doxo replicates). (**I**) Schematic depiction of senescence induction in hVSMCs causing increased complement and coagulation protein secretion, which is attenuated by DPP4i treatment as determined by mass spectrometry of secreted proteins in the media.

To gain a deeper understanding of the mechanism by which DPP4 inhibition affects senescent VSMC viability, we considered the rise in DPP4 enzymatic activity with senescence (Figure 1E). We performed mass spectrometry analysis on the conditioned media from proliferating and senescent hVSMCs that were either untreated or treated with DPP4i (Figure 4A). Proteomic analysis revealed that hVSMCs rendered senescent by treatment with either CoCl_2_ or Doxo had a robust SASP profile that was different from that of proliferating hVSMCs, and it surprisingly featured increased secretion of many proteins related to complement and coagulation signaling, such as CFD, C4a, C7, FII, FX, and TIMP3 (Figure 4, B and C). Many studies dedicated to elucidating the SASP have concluded that the specific cell type and stimulus largely dictate the unique composition of secreted proteins (30), and hVSMCs may have a distinct SASP that is principally conserved despite the induction method. The SASP of senescent hVSMCs predominantly comprised proteins related to the coagulation cascade and complement activation pathways, according to comprehensive pathway analysis using the National Center for Advancing Translational Sciences (NCATS) BioPlanet (Figure 4D), and reinforced by Reactome network analysis (https://reactome.org/), which demonstrated an enrichment of pathways associated with hemostasis and platelet signaling (Figure 4E). STRING protein interaction analysis (https://string-db.org/) supported the pathway analyses, demonstrating a dense regulatory network of complement and coagulation factors, as determined by the KEGG database (https://www.genome.jp/kegg/pathway.html) (Figure 4F). Historically, the SASP has been widely characterized as comprising secreted proinflammatory chemokines, cytokines, and growth factors, but has recently been shown to include hemostatic factors as well, although this facet had not been evaluated in hVSMCs (31).

Next, we assessed the impact of DPP4 inhibition on the secreted protein profile of senescent hVSMCs. We focused on the proteins that showed altered abundance after DPP4i treatment in both the CoCl_2_ and Doxo groups to ensure that we captured conserved effects on senescent hVSMCs. In keeping with the above findings (Figure 4C), the levels of many coagulation and complement factors that increased by senescence were reduced after treatment with DPP4i (Figure 4, G and H). These mitigating effects support our hypothesis that DPP4 promotes senescence by cleaving extracellular proteins, triggering their activation and downstream signaling (Figure 4I). A majority of the proteins altered by DPP4i treatment are cleaved and activated throughout the complement or coagulation cascade, and these two pathways often cross communicate to activate or terminate one another (32). While the proteomic data did not conclusively identify a specific protein or set of proteins directly cleaved by DPP4, blunting the enzymatic activity of DPP4 had clear senomorphic properties to weaken the SASP of senescent hVSMCs, which may also contribute to reduced senescent cell viability.

**Figure 5:**
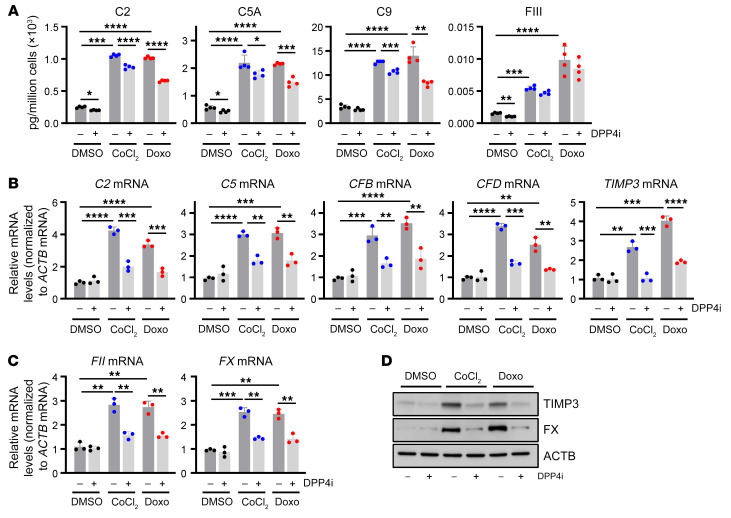
Validation of secreted complement and coagulation proteins in senescent hVSMCs. (**A**) Bioplex analysis of secreted proteins from hVSMCs treated with DMSO, CoCl_2_, and Doxo for 7 days with (+) and without (–) DPP4i treatment. C2, C5A, C9, and FIII were measured (*n* = 3). (**B** and **C**) RT-qPCR analysis of mRNAs encoding complement (**B**) and coagulation (**C**) factors following treatment with DMSO (–) or DPP4i (+) as described in **A**. (**D**) Western blot analysis of the levels of factor X (FX), TIMP3, and loading control ACTB in hVSMCs that were processed as described in **A**. Data are represented as mean ± SD from *n* = 3 or indicated biological replicates. Significance was established using Student’s *t* test with Bonferroni’s correction. *P ≤ 0.05; **P ≤ 0.01; ***P ≤ 0.001; ****P ≤ 0.0001.

To validate the proteomic results, we employed a combination of extracellular and intracellular analyses. Using multiplexed ELISA, we were able to quantify several complement and coagulation factors in the conditioned media from the proliferating and senescent hVSMCs treated with DPP4i. The results were consistent with the mass spectrometry data, demonstrating reductions in most complement factors (C2, C5A, C9) and a coagulation factor (FIII) as a result of DPP4 inhibition (Figure 5A and Supplemental Figure 5A).

**Figure 6:**
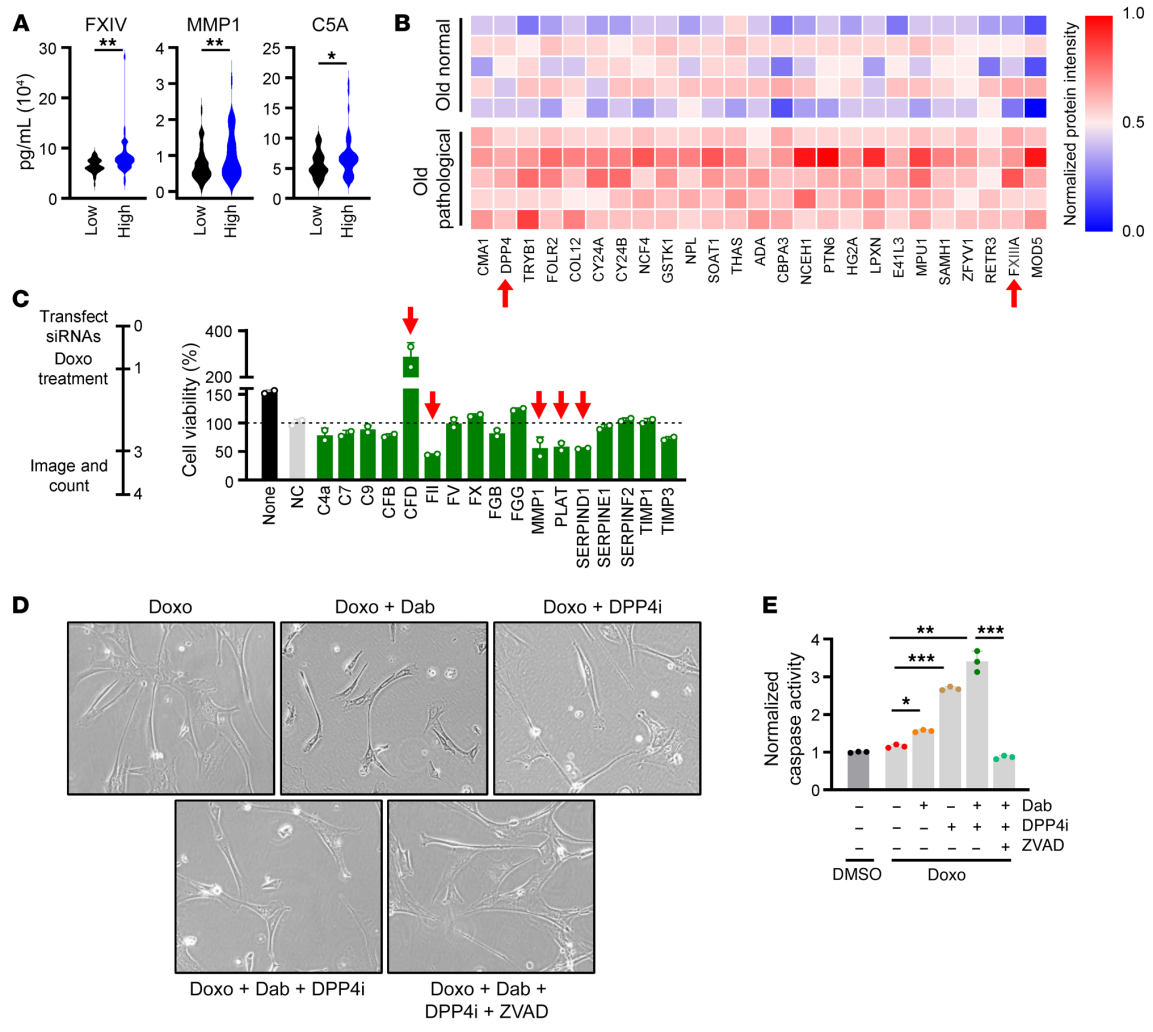
DPP4 downstream targets enable senescent cell viability. (**A**) Bioplex analysis of complement and coagulation proteins in human serum samples from people designated with low and high FRS (*n* = 50 per group). (**B**) Normalized protein intensity represented in a heatmap comparison of normal and pathological (atherosclerotic) aortic tissue from >62-year-old donors. (**C**) Percentage of cell viability graphed for each siRNA transfected into hVSMCs treated with Doxo (*n* = 2). The dashed line indicates 100% viability, and the red arrows point to siRNAs that resulted in approximately 50% reduction or increase in cell viability. (**D** and **E**) hVSMCs were treated with the indicated conditions for 7 days and micrographs were obtained (**D**) and caspase-3 and -7 activity levels were calculated (**E**). Original magnification, ×20. Data are represented as mean ± SD from *n* = 3 or indicated biological replicates. Significance was established using Student’s *t* test with Bonferroni’s correction. *P ≤ 0.05; **P ≤ 0.01; ***P ≤ 0.001.

Despite our expectation that modulating DPP4 would not alter intracellular levels of complement and coagulation factors, we analyzed their expression levels as an indirect consequence of DPP4 silencing or inhibition. Surprisingly, the levels of mRNAs encoding complement and matrix remodeling factors (C2, C5, CFB, CFD, TIMP3) and coagulation factors (FII, FX) were largely elevated by senescence induction, while silencing DPP4 (Supplemental Figure 5, B and C) or treatment with DPP4i (Figure 5, B and C) reduced their abundance. These results suggest that loss or inhibition of DPP4 disturbs autocrine and paracrine signaling that may control the production of complement and coagulation factors in senescent cells. We focused on 2 key factors in the coagulation cascade that were suppressed by DPP4 inhibition in senescent hVSMCs, TIMP3 and FX; FX is critical for FII activation, and FII is central to many downstream signaling pathways such as platelet activation, fibrin clotting, and eventual complement activation (33). As shown by Western blot analysis, the intracellular levels of FX and TIMP3 greatly increased in cells rendered senescent by CoCl_2_ or Doxo, and these elevations were reduced when DPP4 was silenced (Supplemental Figure 5D) or inhibited (Figure 5D). Finally, after confirming that DPP4i conveys senomorphic properties by suppressing complement and coagulation factors secreted by senescent VSMCs, we measured the presence of such proteins in people with low and high FRS from the BLSA. Participants with high risk for cardiovascular disease displayed increased serum levels of several factors secreted by senescent cells, including FXIV, MMP1, and C5A (Figure 6A). These results were bolstered by mass spectrometry analysis of human aortic tissue from older people, where we found that regions with atherosclerotic lesions were enriched for DPP4 and coagulation factor FXIIIA, suggesting that DPP4 is present and potentially functional with age in regions of senescence, despite remaining unchanged in serum (Figure 6B). Together, these results support a role for DPP4 linking cell senescence to complement and coagulation cascades in vascular disease.

### Coagulation factors mediate the senomodulatory effects of DPP4i in senescent hVSMCs.

To elucidate the impact of complement and coagulation factors on the viability and trajectory of senescent hVSMCs, we silenced 17 of the top DPP4 targets identified in our mass spectrometry data using specific siRNAs and screened for changes in cell viability following senescence induction. We imaged and counted cells 48 hours after Doxo treatment, a time frame in which cell-fate decisions between senescence and apoptosis are apparent (34). Silencing 4 coagulation-related factors, FII, MMP1, PLAT, and SERPIND1, significantly reduced cell viability to near or below 50% (Figure 6C and Supplemental Figure 6A). Reducing the levels of complement factors did not immediately affect cell viability, although silencing CFD unexpectedly permitted cells to escape senescence and resume proliferation (Figure 6C). Cell-viability measurements 72 hours after inducing senescence indicated that silencing complement factors required more time to trigger cell death (Supplemental Figure 6B). These results indicate that inhibiting DPP4 has a dual effect; on the one hand, it causes moderate senolysis, and on the other, it reduces the production of downstream coagulation and complement factors.

Given the essential role of thrombin (FII) as an activator of procoagulation and complement pathways and its strong effect on senescent cell viability (Figure 6C), we considered whether its pharmacological inhibition would cause similar or stronger effects than DPP4i. We tested this possibility by incubating proliferating and senescent hVSMCs with dabigatran (Dab), a reversible and selective thrombin inhibitor (35). Simultaneous treatment of Dab with Doxo for 7 days modestly increased cell death (Figure 6D) and reduced expression of FX, a representative of the coagulation factors induced by senescence but suppressed by DPP4 inhibition (Supplemental Figure 6C). While both Dab and DPP4i moderately induced senescent cell death, the combination of both drugs synergistically increased caspase-3 and -7 activity and reduced senescent cell viability. To further confirm that this effect was mediated by apoptosis, we concurrently administered a pan-caspase inhibitor, ZVAD-FMK, along with Dab and DPP4i. The caspase inhibitor rescued senescent cells from apoptosis and reduced caspase activity to near control levels (Figure 6, D and E). These results support the hypothesis that coagulation and complement factors can act locally to enhance the viability of senescent hVSMCs and that inhibiting or removing specific elements of these pathways may improve senolytic efficacy.

### Inhibition of DPP4 in the Ldlr^–/–^ mouse model of atherosclerosis reduces senescent cell burden.

**Figure 7:**
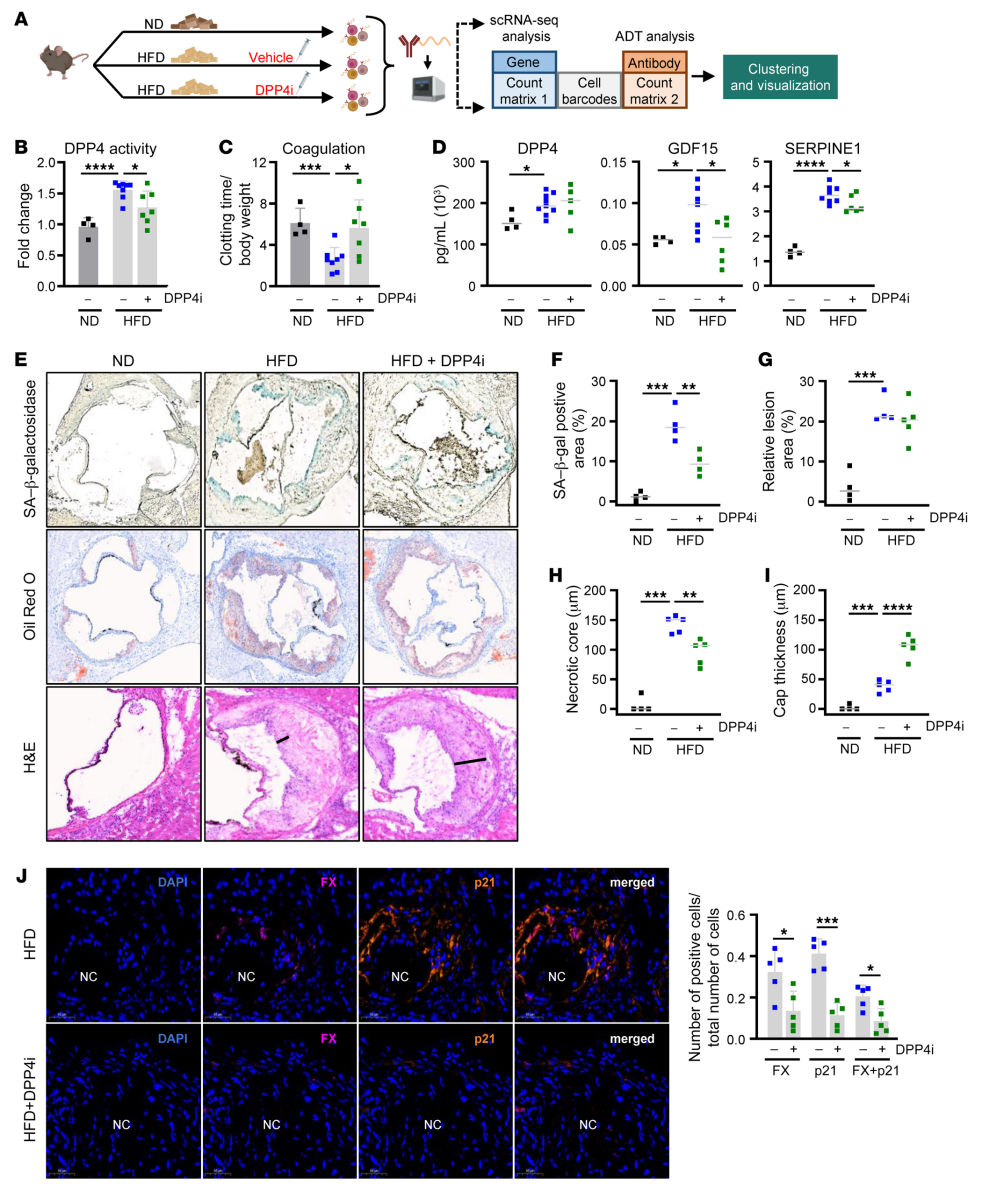
Inhibition of DPP4 in *Ldlr^–/–^* mouse model of atherosclerosis reduces coagulation factors and senescent cell accumulation. (**A**) Diagram of the experimental design for treating *Ldlr^–/–^* mice with ND or HFD with vehicle or DPP4i. Following 16 weeks of diet and treatment, aortas were isolated, digested, and incubated with a DPP4 antibody–derived CITE-Seq tag for scRNA-Seq. (**B**) DPP4 activity levels were measured in serum from *Ldlr^–/–^* mice fed a ND (*n* = 4), HFD (*n* = 6), or HFD+DPP4i (*n* = 6). (**C**) Clotting time was measured in *Ldlr^–/–^* mice fed a ND, HFD, or HFD+DPP4i using tail bleeding as a surrogate. (**D**) Bioplex analysis of circulating DPP4, GDF15, and SERPINE1 in serum from *Ldlr^–/–^* mice fed a regular diet, HFD, or HFD+DPP4i. (**E**) Representative images (*n* = 4–5 per condition) of aortic roots from ND-, HFD-, and HFD+DPP4i–treated mice stained with SA–β-gal (top), Oil Red O (middle), and H&E (bottom). Original magnification, ×100. (**F**) Quantification of SA–β-gal–positive area. (**G**) Quantification of relative lesion area. (**H**) Quantification of necrotic core thickness based on H&E staining. (**I**) Quantification of cap thickness (black line) based on H&E staining. (**J**) Left: immunofluorescent analysis of aortic roots with DAPI (blue), FX (purple), and p21 (orange) signals. Necrotic core area indicated by NC. Right: percentage of FX, p21, or double-positive (FX+p21) cells in HFD or HFD+DPP4i mice (*n* = 5). Original magnification, ×40. In **E**, H&E HFD image is from a different crop of SF6E HFD. Significance was established using Student’s *t* test or 1-way ANOVA via Tukey’s HSD. *P ≤ 0.05; **P ≤ 0.01; ***P ≤ 0.001; ****P ≤ 0.0001.

In light of previous findings that treatment of atherosclerotic mice with DPP4 inhibitors such as anagliptin had beneficial effects on disease progression, we sought to expand our investigation to a mouse model (29). Importantly, in some studies, the positive effects of these drugs are completely independent of DPP4’s canonical role in glucose metabolism (13), highlighting the need to explore other mechanisms by which DPP4 functions in atherosclerotic vascular diseases. To determine the effect of DPP4 inhibition on senescent cells in atherosclerosis, we adopted the atherosclerosis model of *Ldlr^–/–^* mice fed a HFD and simultaneously administered DPP4i or a vehicle control by oral gavage 5 days per week for 16 weeks or fed a ND (Figure 7A). To address the effects of DPP4i, we first measured DPP4 activity in the serum; as expected, DPP4 activity increased with HFD, but was significantly reduced upon treatment with DPP4i (Figure 7B). We next measured the effect of DPP4i on coagulation by measuring the tail-bleeding time to cessation. Strikingly, atherosclerotic animals (HFD) had faster clotting times compared with control mice (ND), and treatment with DPP4i rescued this result, increasing tail-bleeding times significantly (Figure 7C). Inhibiting DPP4 activity did not change the abundance of circulating DPP4 in serum, although it did reduce the levels of GDF15, a conserved SASP factor and cardiovascular disease biomarker that increased with HFD. Importantly, the levels of SERPINE1, another conserved SASP factor and coagulation component that was most significantly elevated in atherosclerotic mouse serum in our panel, was also reduced by DPP4i treatment (Figure 7D). While other SASP factors did not change significantly, TIMP1, MMP12, CXCL1 (a known DPP4 target in mice), CCL3, and VEGF all displayed a similar trend, increasing with HFD and declining with DPP4i treatment (Supplemental Figure 6D) (36). These findings reinforce the senomorphic properties of DPP4i and provide evidence that the global beneficial effects of DPP4i are not limited to the local atherosclerotic niche.

Next, we evaluated aortic root sections for morphology by H&E staining, SA–β-gal activity, and lipid deposition (Oil Red O) (Figure 7E). As a surrogate marker of senescence, SA–β-gal activity increased with HFD and declined with DPP4i treatment (Figure 7F). Although we did not find reductions in atherosclerotic plaque burden in mice in the HFD group when comparing treatment with DPP4i relative to no treatment, as assessed by staining with Oil Red O (Figure 7G), there was an important reduction in the necrotic core thickness and area as well as an increase in cap thickness after DPP4i treatment, as measured by H&E staining (Figure 7, H and I and Supplemental Figure 6E). These findings suggest that DPP4 and its downstream targets may exert their influence on plaque stability rather than plaque buildup. This possibility was further supported by immunofluorescent staining of the aortic roots, where we observed that mice in the HFD group displayed increased signals for senescence marker p21 (40%) and for FX (32%) as well as double-positive (20%) cells around necrotic core areas, while DPP4i-treated mice featured fewer p21 (11%), FX (13%), and double-positive (8%) cells (Figure 7J). These data show that DPP4i reduces circulating SASP coagulation factors SERPINE1 and GDF15, necrotic core thickness and area, and the percentage of senescent cells harboring coagulation factors (p21- and FX-positive cells) around the necrotic core, all of which contribute to plaque vulnerability (37–40). These results support our hypothesis that DPP4i improves plaque stability in mouse atherosclerosis by suppressing senescence-associated coagulation.

### Inhibition of DPP4 in the Ldlr^–/–^ mouse model of atherosclerosis alters the composition of aortic cells.

**Figure 8:**
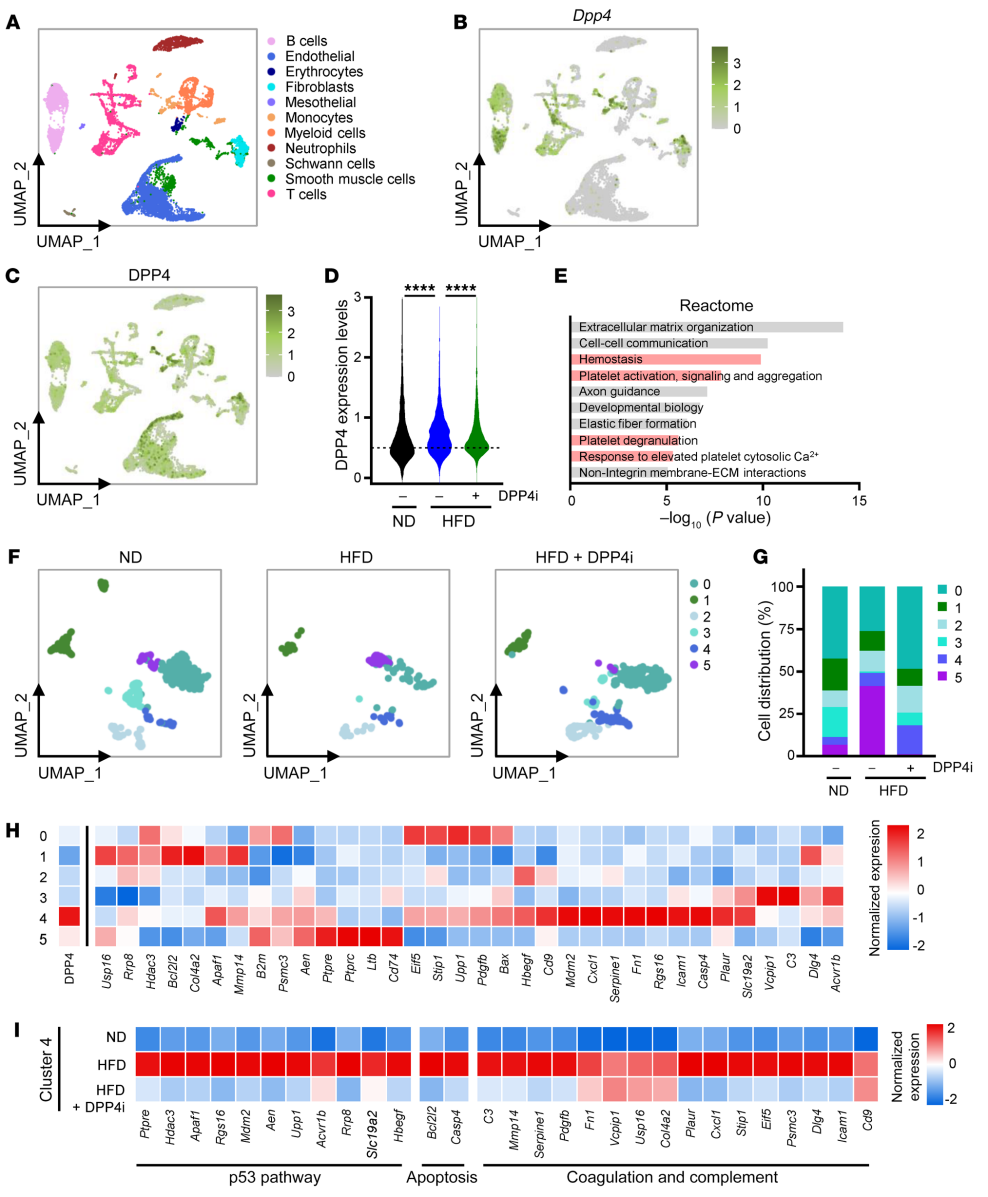
DPP4 CITE-Seq analysis of *Ldlr^-/-^* mouse model of atherosclerosis treated with DPP4i. (**A**) Uniform Manifold Approximation and Projection (UMAP) plot of 16,050 aortic cells from all the samples color labeled by major cell types. (**B**) *Dpp4* mRNA distribution across all aortic cell types. (**C**) DPP4 cell-surface protein expression across all aortic cell types. (**D**) DPP4 protein expression levels in mice fed an ND, HFD, or HFD+DPP4i (*n* = 4, each condition). (**E**) Reactome pathway analysis of transcripts with positive correlation (*R* >0.2) to all aortic cells with DPP4 cell-surface protein. (**F**) UMAP of VSMC subset reclustered in ND, HFD, and HFD+DPP4i. (**G**) Composition (%) of VSMC subclusters in ND, HFD, and HFD+DPP4i. (**H**) Heatmap of DPP4 protein (far left) and mRNAs enriched in VSMC subclusters 0 through 5. (**I**) Heatmap of key cluster 4 mRNAs differentially expressed in ND, HFD, and HFD+DPP4i, categorized by p53 pathway-, apoptosis-, coagulation-, and complement-related genes. ****P ≤ 0.0001.

Next, we assessed the composition of cells in the aorta as a result of DPP4 inhibition using single-cell RNA sequencing (scRNA-Seq) and DPP4 cellular indexing of transcriptomes and epitopes by sequencing (CITE-Seq) analyses (41). We identified 11 cell types defined by 21 clusters of aortic cells using cell type–specific markers for all major cell types as defined in the literature (42–45) (Figure 8A, Supplemental Figure 7 and Supplemental Figure 8, A and B). We visualized total *Dpp4* mRNA expression levels as well as the levels of DPP4 protein distribution on the cell surface in all cells (Figure 8, B and C). Surprisingly, HFD did not increase the levels of DPP4 expressed on the cell surface per cell, but instead it increased the number of cells expressing DPP4 on their surface; treatment with DPP4i decreased the number of DPP4-positive cells (Figure 8D). Reactome pathway analysis of transcripts with positive correlation to all DPP4-positive cells (*R* > 0.2) revealed that 4 of the top 10 pathways were associated with hemostasis and platelet activation; other relevant pathways included extracellular matrix organization and cellular communication, also related to DPP4 signaling in senescent cells (Figure 8E). Inhibition of DPP4 greatly influenced the composition of cells in the aorta, reducing the immune cell populations and restoring cells that are vital for arterial homeostasis (i.e., VSMCs, endothelial cells, and fibroblasts) (Supplemental Figure 8C). These results confirm a general antiinflammatory effect previously observed in atherosclerotic mice treated with DPP4i (46).

DPP4 is ubiquitously expressed in many cell types, especially T and B cells (22, 47). To further clarify the role of DPP4, we analyzed the VSMC clusters to determine subpopulations altered by HFD and DPP4i treatment (Figure 8, F and G and Supplemental Figure 9A). Combining and reclustering all VSMCs unveiled 6 subclusters defining VSMC subtypes (Figure 8H and Supplemental Figure 9, A and B). Within these subclusters, cluster 4 exhibited the highest expression and largest number of DPP4-positive cells, and cluster 5 also demonstrated DPP4 cell-surface enrichment (Figure 8H, far left on heatmap). Despite a lack of reduction in cell number by DPP4i treatment, cluster 4 featured transcriptomic patterns consistent with senescence and coagulation, including those related to p53 signaling (*Mdm2*, *Upp1*, and *Ptpre* mRNAs) and cell survival (*Casp4* and *Bcl2l2* mRNAs). Even more striking was the increased expression of mRNAs encoding complement and coagulation factors (*C3*, *Mmp14*, *Serpine1*, and *Fn1* mRNAs). Consistent with our VSMC culture model, treatment with DPP4i significantly reduced the expression levels of many mRNAs encoding proteins with roles in complement system and coagulation, cell survival, and p53-related actions that were increased in the HFD mice, strengthening the supposition that DPP4i possesses senomorphic properties to suppress the production of important SASP factors (Figure 8I and Supplemental Figure 9C). Cluster 5 was increased in HFD and reduced by DPP4i treatment and was the second of 2 VSMC clusters with enhanced DPP4 surface-protein expression (Figure 8G). Gene expression analysis of cluster 5 revealed a unique phenotype lacking many of the traditional smooth muscle contractile markers, such as *Acta2*, *Tagln*, and *Myh11* mRNAs, a trait shared with dedifferentiated VSMCs, and increased expression of *Cd74*, *Ptprc*, *Ltb*, and *B2m* mRNAs, which encode proteins associated with T cell recruitment and monocyte activation (Figure 8H). Importantly, both *Cd9* mRNA (cluster 4) and *B2m* mRNA (cluster 5) were associated with senescence, underscoring the heterogeneity of senescent cells even within a single cell type such as VSMCs (48, 49).

In summary, CITE-Seq single-cell analysis uncovered 2 VSMC subclusters expressing higher levels of DPP4 on the cell surface, and each cluster comprised a unique transcriptome associated with senescence and hemostasis (cluster 4) or inflammation and immune cell recruitment (cluster 5). DPP4i affected differently each potentially senescent DPP4-positive cluster: it strongly reduced cell numbers in cluster 5, while it suppressed complement, coagulation, and p53-related gene expression in cluster 4. The results indicate that DPP4 inhibition is not strictly senolytic or senomorphic, but rather perturbs the senohemostasis maintained by DPP4. Accordingly, DPP4i curbs the complement and coagulation cascades to induce moderate senolysis and to promote beneficial gene expression in VSMCs during atherosclerosis.

## Discussion

While senescent cells possess beneficial functions in certain contexts, such as wound healing or development, there is overwhelming evidence that the accumulation of senescent cells in age-related diseases contributes to disease development or progression (50). As a result, many studies have begun to explore therapeutic avenues targeting senescent cells (senotherapies) to improve the aging process (51). In mouse models of atherosclerosis, selective removal of p16-expressing senescent cells or senolytic treatments improved plaque burden in the aorta, suggesting that senescent cells participate in key aspects of atherosclerosis (40, 52–54). The role of DPP4 in metabolic and age-related diseases originated with the discovery that this exopeptidase selectively cleaves N-terminal dipeptides, inactivating GLP-1 and thereby restoring glucose homeostasis and improving type 2 diabetes (18, 19). Subsequently, DPP4 was implicated in alleviating metabolism-related diseases such as obesity, chronic liver disease, and atherosclerosis (55–58). Interestingly, DPP4 inhibitors have been proven effective at reducing atherosclerosis in mice independently of their canonical impact on glucose metabolism, suggesting that DPP4 has important functions in nonmetabolic tissues and cells (13, 59). As mentioned, recent studies have identified DPP4 on the surface of senescent cells, and soluble DPP4 was found to trigger endothelial senescence, demonstrating that DPP4 may contribute to the development of the senescent phenotype (11, 59). In cultured cells, we found that inhibiting DPP4 on the surface of senescent VSMCs suppressed complement and coagulation programs that foment cell survival, while in mice, inhibiting DPP4 generally reduces the presence of senescent cells and factors that contribute to plaque vulnerability. A key result from our study is that downstream targets of DPP4 inhibition are elevated in people with high FRSs (Figure 6A); accordingly, future investigation of whether complement and coagulation factors are effective therapeutic targets for improving vascular disease outcomes is warranted.

In our study, the levels of *DPP4* mRNA and whole-cell and surface DPP4 protein increased in senescent hVSMCs. This rise was supported by augmented DPP4 enzymatic activity in senescent hVSMCs as well as in serum from atherosclerotic mouse and monkey models with increased senescent cell populations in tissues. Moreover, DPP4 protein abundance was also elevated in aged human, mouse, and monkey aortic tissue (Figure 1, G–L, and Supplemental Figure 2, B and C), suggesting that DPP4 increases in atherosclerosis, conditions often associated with increased senescent cells. Surprisingly, knockdown or inhibition of DPP4 in cultured senescent hVSMCs elevated caspase-3 and -7 activity and led to increased death of senescent cells (Figure 2, C and F). While DPP4 inhibition does not appear to be strongly senolytic, it did moderately lower the viability of senescent cells (Figure 2E), but failed to reduce any senescent cell markers, including SA–β-gal activity or senescence-associated proteins.

Given its consistent elevation in atherosclerosis and the negative impact of DPP4 silencing on senescent cell viability, we sought to determine the role of DPP4 on the surface of senescent cells. DPP4 has unique peptidase activity, leading us to hypothesize that its ability to cleave proteins might be an integral facet of the senescence program. Mass spectrometry analysis of conditioned media from senescent hVSMCs uncovered a conserved pattern of SASP factors that comprised complement and coagulation factors. A previous study identified increased hemostasis factors in the SASP of senescent fibroblasts (31), but these factors had not been validated in other paradigms of senescence. Recently, coagulation factor IX was implicated as a regulator of senescence in cancer cells and inhibition of coagulation factor Xa induced plaque regression and increased plaque stability (60, 61). The secretion of procoagulation and complement proteins has particular relevance in vascular diseases in which thrombosis and plaque vulnerability critically increase the risk of mortality (61, 62); therefore, targeting senescent cells in the atherosclerotic niche may reduce the probability of thrombosis or plaque rupture, as previously demonstrated (10). Importantly, treatment of senescent hVSMCs with DPP4i substantially reduced or prevented the secretion of complement and coagulation proteins, while individual silencing of many coagulation and complement factors induced senescent cell death. These results indicate that inhibiting DPP4 may have senomorphic effects to suppress the SASP of senescent hVSMCs, consequently altering cell communication and cell viability.

It remains unclear whether DPP4 directly cleaves complement and coagulation proteins, despite the finding that this group of proteins was highly secreted by senescent VSMCs and strongly reduced by DPP4i treatment. In our mass spectrometry data, we only identified a small number of potential DPP4 targets based on a putative penultimate alanine, proline, or serine in the DPP4 truncation site. Of the potential DPP4 targets, we observed no change in CCL26, increased secretion of IL-1α and IL-1β, which was not statistically significant, and increased secretion of LTF and TF by senescent VSMCs; however, TF was unaffected by DPP4i treatment (36). While previous studies have identified chemokines and cytokines secreted by senescent VSMCs, such as IL-1α and IL-6 (3), very few studies have measured secreted proteins in an unbiased manner. Although we may expect that senescent VSMCs secrete many chemokines, cytokines, and growth factors, our results identified complement and coagulation factors as highly secreted, and this may partly reflect the chosen senescence inducers, the time of collection, and the method of detection. Further, many cleavage targets of DPP4 remain unknown, as the cleavage event is small and occurs at dipeptides on the N-terminus, suggesting that our data may help to uncover new proteolytic targets of DPP4.

To evaluate a possible translational angle for these findings, we measured complement and coagulation proteins in the serum of participants from the BLSA aged 50 to 65, with high and low FRS as measures of cardiovascular risk. While circulating DPP4 was unchanged, MMP1, C5A, and coagulation factor XIV were all significantly increased in people with high FRS, while DPP4 and FXIIIA proteins increased in atherosclerotic tissue from older people (Supplemental Figure 2F and Figure 5A). Even though DPP4 in these participants was not a strong circulating indicator of cardiovascular risk, participants with high FRS displayed an interesting rise in proteins downstream in the DPP4 cleavage cascade; these proteins represent potential biomarkers of cardiovascular disease and senescence via the SASP. Therefore, these and other proteins related to the complement and coagulation cascades may also serve as therapeutic targets for improving cardiovascular disease and suppressing or removing senescent cells. In particular, C5A has been implicated as a predictor of future cardiovascular events in patients with advanced atherosclerosis, and MMPs are considered biomarkers of plaque instability in atherosclerosis, suggesting that senescent cells may be partially responsible for the secretion of these factors (63, 64). Importantly, while the serum samples from this BLSA cohort included approximately 50 males and approximately 50 females, the females were generally healthier with less disparity in risk score.

We sought to recapitulate previous studies in which DPP4 inhibitors were effective at reducing atherosclerosis and inflammation in mice (29). *Ldlr^–/–^* mice fed an HFD experienced increased DPP4 activity and reduced tail-bleeding time relative to body weight compared with mice fed a ND, and treatment with DPP4i restored the coagulation phenotype and lowered DPP4 activity. These results support the notion that the accumulation of senescent cells during atherosclerosis may contribute to increased production of coagulation proteins and to triggering thrombosis. Serum factors such as GDF15 and SERPINE1, both biomarkers of cardiovascular disease and highly conserved SASP factors, were increased with HFD and reduced with DPP4i treatment (30, 65, 66). Perhaps most importantly, when we assessed plaques in the different groups, we observed that DPP4i treatment significantly reduced the thickness of the necrotic core, while increasing cap thickness (Figure 7, E, H, and I and Supplemental Figure 6E). These results suggest that, through its proteolytic activity, DPP4 enhances plaque vulnerability and that different doses, times, or inhibitor types could improve the therapeutic effect we observed. We evaluated the presence of the senescence marker p21 and the coagulation factor FX in the aortic root and found colocalization of the 2 signals in approximately 20% of cells concentrated to areas surrounding the necrotic core; treatment with DPP4i decreased these double-positive cells to approximately 8%. Altogether, DPP4i reduced senescent cells and their secretion of coagulation factors that contribute to plaque instability and the risk of plaque rupture.

To understand in greater depth the role of DPP4 and the impact of DPP4i on aortic cells, we performed CITE-Seq analysis. Given that DPP4 is expressed on the surface of many cells, including T cells, we employed single-cell analysis to understand the effect of DPP4 on individual cell types, particularly VSMCs. We found an increased number of cells displaying DPP4 on the surface of VSMCs in mice fed the HFD, and this population was reduced by DPP4i treatment. When we examined the transcriptome of all DPP4-expressing cells using Reactome pathway analysis, we encountered many pathways related to hemostasis, cell-cell communication, and platelet activation. A deeper look at aortic VSMCs further uncovered 2 unique, potentially senescent subpopulations with increased cell-surface DPP4 expression, clusters 4 and 5. Cluster 4 had the highest DPP4 cell-surface expression among VSMCs and displayed characteristically senescent markers related to the p53 program and cell survival as well as complement and coagulation factors. Cluster 5 had elevated cell-surface DPP4, and its transcriptome was suggestive of dedifferentiation into inflammatory, potentially senescent VSMCs that were responsive to HFD and suppressed by DPP4i. While cluster 5 either did not develop or was selectively killed by DPP4i, cluster 4 showed strong suppression of the p53, survival, complement, and coagulation programs by DPP4i. Future studies focused on the contribution of these cells to the development and progression of atherosclerosis are warranted in order to develop senohemostatic drugs to alleviate disease burden.

To date, DPP4 inhibitors have not been effective in clinical studies to improve cardiovascular outcomes (67); however, many animal studies have demonstrated reduced atherosclerotic burden and inflammation in animals treated with these drugs (29). This discrepancy may be attributed to organismal differences such that these relatively small beneficial effects may not translate well to humans; at the same time, the proinflammatory effects of DPP4 may be elicited at an early stage that promotes disease development, but may be less influential if treatment is administered at later stages (68, 69). Other factors could include the time and duration of the intervention, dose, and type of inhibitor. A recent study found that DPP4 inhibitors directly suppressed the functional activity of the lectin complement pathway, independently of DPP4, suggesting that DPP4 inhibitors can suppress the SASP of any cell-secreting complement factors (70). While the search for successful senolytic drugs continues, combination therapies that include senolytics and senomorphics have been suggested; it is possible that DPP4 inhibitors could be used in a similar manner (71). Additionally, our data suggest that the downstream proteins reduced by DPP4 inhibition hold greater promise as biomarkers or therapeutic targets. Perhaps focusing on proteins downstream of DPP4 will improve specificity, permitting us to avoid inhibiting proteins that instead promote cell survival, such as CFD.

Overall, the results from this study provide an area of investigation for reducing or suppressing senescent vascular cell function to improve the burden of atherosclerosis that is maintained through senohemostasis. We propose the use of drugs that both suppress the SASP and selectively kill senescent cells by targeting hemostastic pathways of complement and coagulation. We have also identified a SASP program associated with VSMCs that illuminates how senescent cells communicate with neighboring cells and tissues to promote disease development. We hypothesize that proteins downstream of DPP4 represent potential biomarkers of cardiovascular disease that inform on the senescent cell burden and constitute viable therapeutic targets.

## Methods

### Cell culture, senescence induction, and SA–β-gal activity.

hVSMCs were obtained as cryopreserved secondary cultures from LifeLine Cell Technology and maintained in VascuLife SMC Medium Complete Kit from LifeLine Cell Technology following the manufacturer’s protocol. hVSMCs were rendered senescent by treatment with 125 nM doxorubicin (Doxo) or 200 nM CoCl_2_ (hypoxia mimic) for 7 to 10 days. For DPP4i treatment in culture, 10 to 20 nM of DPP4i (Selleckchem) or 20 nM of Dab (Selleckchem) was added simultaneously with senescence induction or DMSO and refreshed every 3 days. SA–β-gal activity was assessed using the Senescence β-Galactosidase Staining Kit (Cell Signaling Technology). Human WI-38 cells, obtained from the NIGMS Human Genetic Cell Repository at the Coriell Institute for Medical Research (repository ID AG06814-N), and HRECs (ATCC) were cultured in DMEM (Gibco; Thermo Fisher Scientific) supplemented with 10% heat-inactivated FBS (Gibco; Thermo Fisher Scientific) and renal epithelial cell basal medium prepared according to the manufacturer’s instructions, respectively, each supplemented with 0.5% penicillin-streptomycin (Gibco; Thermo Fisher Scientific), sodium pyruvate (Gibco; Thermo Fisher Scientific), and nonessential amino acids (Gibco; Thermo Fisher Scientific) and cultured under the same conditions (5% CO_2_). Cells were maintained at low population-doubling levels (PDL) for the experiments included in this study.

### siRNAs and drug treatments.

For TF silencing, hVSMCs were transfected with siCtrl or siRELA, siEGFR, siSP1, or siFOS (25 nM) (Dharmacon); 72 hours later, senescence was induced with Doxo for an additional 72 hours. For DPP4 silencing, hVSMCs were transfected with siCtrl or siDPP4 (25 nM). For siRNA screening, a custom siRNA library plate was designed including siRNA pools to silence CFD, C9, CFB, C4A, C7, FGB, FGG, FX, FV, FII, SERPINE1, TIMP1, MMP1, and PLAT. This included a nontargeting control siRNA. Sequences and catalog numbers for siRNA custom screening are available in Supplemental Table 1. All small RNA transfections were performed using Lipofectamine RNAiMAX (Thermo Fisher Scientific).

### Caspase-3 and -7 activity.

Apoptosis was monitored by measuring caspase-3 and -7 activity using the Caspase-Glo 3/7 Assay System (Promega) as previously described (72).

### RNA isolation and RT-qPCR analysis.

RNA isolation and RT are described in detail in the Supplemental Methods. PCR primers are listed (Supplemental Table 2).

### Mouse cell-surface analysis.

A single-cell suspension of mouse aortic cells was prepared as described below (Single-cell preparation and scRNA-Seq and CITE-Seq analysis). Cells were resuspended in 100 μL of resuspension buffer (DMEM supplemented with 15% FBS) and incubated with 5 μL of mouse TruStain FcX Fc blocking reagent (BioLegend) for 5 minutes, followed by a 15-minute incubation with an antibody cocktail consisting of 5 μL of anti-mouse CD26-APC (BioLegend, clone H194-112), 5 μL of anti-mouse CD31-FITC (BioLegend, clone 390), 3 μL of anti-mouse CD45-PE/cyanine 7 (BioLegend, clone 30-F11), and 5 μL of anti-mouse TER119–Pacific Blue (BioLegend, clone TER-119) at 25°C in the dark. Cells were then washed with 1 mL of resuspension buffer and spun at 400*g* for 5 minutes; after removing the supernatant, cells were resuspended in 1 mL of resuspension buffer supplemented with 2 μg/mL of PI and 5 mM EDTA. DPP4-positive VSMCs were identified as TER-119^–^CD45^–^CD31^–^CD26^+^ (73, 74) and sorted directly into TRIzol LS Reagent using FACS Aria FUSION (BD Bioscience) for analysis.

### hVSMC mass spectrometry and proteomics.

hVSMCs were seeded in T75 flasks and treated with DMSO, CoCl_2_, or Doxo as well as incubated with DPP4i every other day, also in triplicate. After 7 days, we replaced the media with 6 mL of media that contained DPP4i or DMSO and did not have FBS; 16 hours later, the media were collected and centrifuged at 1,000*g* for 10 minutes to remove debris. Each replicate contained approximately 6 × 10^6^ cells, and cell pellets and media were stored at –80°C until processing. To process the samples, media collected were concentrated using a SpeedVac (Thermo Fisher Scientific) until a dry pellet was observed. Mass spectrometry of conditioned media was performed and analyzed as described (75); data are available in Supplemental Table 3.

### Media and serum collection.

Media from cultured cells were collected and centrifuged at 16,000*g* for 4 minutes at 4°C. A total of 50 μL of the supernatant was added to each well for analysis. Mouse serum was collected and allowed to clot for 2 hours at 25°C. Samples were then centrifuged for 20 minutes at 2,000*g*. Serum was removed and frozen at –80°C. Duplicate samples were prepared. Human serum was collected as previously described (76).

### Human aortic tissue preparation and staining.

Segments of abdominal aortas were procured within 24 to 48 hours of patients dying from noncardiovascular deaths due to unnatural causes (homicide, suicide, or accident). Grossly normal intima and media of 15 White males divided into 2 age groups (young: 33.2 ± 2.2 yr, *n* = 15; old: 73.8 ± 4.3 yr, *n* = 15) were obtained at autopsy, over a 2-year period (July 2001 to August 2003), from the Department of Cardiovascular Pathology, Armed Forces Institute of Pathology (Silver Spring, Maryland, USA). The protocol was modified as previously described (77).

### Human aortic proteomics.

Archival human abdominal frozen aortic samples were collected from autopsies of 5 male subjects aged 62 to 72 years old dying of unnatural causes (homicide, suicide, or accident) and noncardiovascular deaths. Specimens were harvested 4 to 8 hours after death (78). Aortic wall with less than 10% fatty streaks or fatty dots on the luminal surface and no protruding plaque was considered as normal, and an atherosclerotic plaque was defined as an aortic wall with 2 or more protruding lesions observed under the macroscope or by the naked eye (78, 79). Tissue regions selected to represent normal aorta and atherosclerotic plaque were prepared for extraction of protein for proteomic analysis. Mass spectrometry and analysis were performed by Poochon Scientific (Supplemental Table 4).

### Mouse studies.

LDL receptor knockout (*Ldlr*^−/−^) mice on the C57BL/6 background purchased from Jackson Laboratory (catalog 002207) were bred to obtain homozygous *Ldlr*^−/−^ KO mice. Mice of both sexes were entered in the study at 8 weeks of age when normal chow was replaced with an atherogenic diet (42% fat, 0.2% cholesterol, Harlan Atherogenic Diet TD.88137). For the treatment groups, mice were gavaged with 10 mg/kg/d of DPP4i from SelleckChem or an equal volume of DMSO, 5 days per week for 16 weeks. Clotting/bleeding time was assessed as previously described (31). C57BL/6 mice were used for the assessment of young (3 m.o.) and old (30 m.o.) mouse aortic VSMCs.

### Atherosclerotic lesion analysis.

Atherosclerotic plaque burden and necrotic core thickness, area, and cap thickness were measured by Oil Red O and H&E staining, as described (80–84). Lesion analysis details are available in the Supplemental Methods.

### Single-cell preparation and scRNA-Seq and CITE-Seq analysis.

Mice were anesthetized and blood was extracted using cardiac puncture. The whole aorta was collected after left ventricular perfusion with 10 mL of PBS and quickly transferred to cold PBS. To prepare a single-cell suspension, perivascular adipose tissue was removed and whole aortas from 3 mice were cut into approximately 1 mm pieces and digested with an enzyme solution consisting of 10 mg/mL collagenase II (MilliporeSigma) and 1 mg/mL elastase (Worthington Biochemical Corp.) for 15 minutes at 37°C. The cell suspension was strained through a 40 μm filter and centrifuged at 500*g* for 5 minutes. Cells were resuspended in 100 μL Cell Staining Buffer (BioLegend) with 15% FBS and 5 μL of mouse TruStain FcX Fc blocking reagent (BioLegend). Cells were incubated for 10 minutes at 4°C, followed by the addition of 1 μg of DPP4 (CD26) APC antibody and incubation for 30 minutes at 4°C. Cells were incubated with TotalSeq APC secondary antibody (BioLegend) for 30 minutes at 4°C. Next, cells were washed with 1 mL of Cell Staining Buffer, spun at 400*g* for 5 minutes, and resuspended in 1 mL of PBS containing 5 mM EDTA. Between 5,000 and 10,000 viable cells were sorted using FACSAria FUSION (BD Bioscience) for 10× Genomics library preparation. The single-cell libraries were prepared with Chromium Next GEM Single Cell 3′ Kit, version 3.1, using the Chromium Next GEM Chip G Single Cell Kit according to the manufacturer’s protocol with Chromium Controller (10x Genomics). The libraries were sequenced with Illumina Nova sequencer at a depth of 70,000–120,000 reads per cell.

scRNA-Seq data for both mRNA and ADT libraries were processed following the Feature Barcoding tool pipeline in Cell Ranger (version 6.1.1; 10x Genomics) and the mouse mm10 reference genome. The obtained read count matrices were subsequently analyzed in R using the Seurat package, version 4.1.0 (85), with default parameters in all functions, unless otherwise specified. A detailed description of the downstream analysis is included in Supplemental Methods.

### Resources.

Details of materials used in this paper, including antibodies, chemicals, commercial assays, cell lines, software, and algorithms, are in the Key Resources Table (Supplemental Table 5).

### Data availability.

Mass spectrometry raw files were uploaded to the repository Chorus (https://chorusproject.org/) (project number 1783). RNA-Seq data were deposited in the NCBI’s Gene Expression Omnibus database (GEO GSE209525).

### Statistics.

Data are represented as mean ± SEM. Significance was determined using a 2-tailed Student’s *t* test with *P* value correction for multiple comparisons (Bonferroni’s correction) when applicable or 1-way ANOVA with individual comparisons via Tukey’s honestly significant difference (HSD) when appropriate. *P* < 0.05 was considered significant.

### Study approval.

All mouse experiments, including the import, housing, experimental procedures, and euthanasia, were performed strictly under an Animal Study Proposal (ASP 474-LGG-2023) and associated amendments and were reviewed and approved by the NIA Animal Care and Use Committee (ACUC). For human samples, this research activity is designated exempt (no. 3353) by the Office of Human Subjects Research (OHSR) and is entered in the OHSR database of the NIH.

## Author contributions

ABH and MG conceptualized the study. ABH, DT, CA, SS, KA, and SD designed experiments. ABH, KMM, AEC, JMG, CAHS, SCH, JZ, RM, JLM, YP, JF, and MED performed and analyzed experiments. MW, MM, JAM, RWM, TT, AZM, YL, LF, RDC, and EGL provided samples (human, mouse, monkey) and analyzed data. ABH and MG wrote the manuscript.

## Supplementary Material

Supplemental data

Supplemental table 1

Supplemental table 2

Supplemental table 3

Supplemental table 4

Supplemental table 5

## Version 1 04/25/2023

In-Press Preview

## Version 2 06/15/2023

Electronic publication
