# Supplementary material for: DPP4 inhibition impairs senohemostasis to improve plaque stability in atherosclerotic mice
Source: J Clin Invest. 2023 Jun 15;133(12):e165933. doi: 10.1172/JCI165933 (PMC10266795; doi:10.1172/JCI165933)
Supplement: Supplemental data [file jci-133-165933-s215.pdf]

SUPPLEMENTAL METHODS

Cellular senescence induction – additional details

Cellular senescence was triggered by different means. Etoposide-induced senescence was achieved by culturing WI-38 and HREC cells for 8-10 days in the presence of etoposide (Selleckchem) at 50 and 20 μM, respectively. To achieve senescence by exposure to ionizing (γ) radiation (IR), WI-38 cells were exposed to 15 Gray (Gy) and cultured for an additional 8-10 days. Oxidative stress-induced senescence was achieved by adding 0.75 mM H_2O_2 directly to WI-38 fibroblasts in complete medium and replacing fresh medium 2 h later; cells were then assayed after 8 days. Media with or without drugs were refreshed every 48 h. Cell viability was assessed by direct cell counting; all cell counts were performed manually by using ImageJ and performed in at least 3 independent replicates. From each replicate, 3 fields were randomly selected and counted. Cell viability was represented as the percentage of remaining cells compared to the number of cells present in the control conditions at the same time.

RNA isolation and RT-qPCR analysis – additional details

RNA was isolated from cells using the TriPure isolation reagent (Roche) and the Direct-zol Mini Kit (Zymo Research) following the manufacturers' protocols and including a step of digestion with DNase I. Total RNA was reverse-transcribed (RT) into cDNA using Maxima reverse transcriptase (Thermo Fisher Scientific) and random hexamers and analyzed by quantitative (q)PCR analysis using SYBR Green mix (Kapa). The relative mRNA expression levels were calculated by the $2^{-\Delta\Delta Ct}$ method and normalized to *ACTB* mRNA levels.

Western blot analysis

Protein extracts were obtained by lysing cells with a denaturing buffer containing 2% sodium dodecyl sulfate (SDS) (Sigma-Aldrich) in 50 mM HEPES. After boiling and sonication, whole-cell protein extracts were size-separated through polyacrylamide gels and transferred to nitrocellulose membranes (Bio-Rad). Membranes were blocked with 5% non-fat dry milk and immunoblotted. Specific primary antibodies were used that recognized DPP4 (Cell Signaling, 67138), LMNB1 (Abcam), TP53 (p53), p21 (CDKN1A) (Cell Signaling), Coagulation Factor X (FX) (Abcam), TIMP3 (Abcam), BCL2L1 (Cell Signaling), BCL2L2 (Cell Signaling), VDAC1 (Santa Cruz Biotechnology), HSP90 (Santa Cruz Biotechnology), and β-actin (ACTB) (Santa Cruz Biotechnology). After incubation with the required secondary antibodies conjugated with horseradish peroxidase (HRP, Jackson Immunoresearch), the chemiluminescent signals were detected by using the Chemidoc system (Bio-Rad).

Human cell surface analysis and DPP4 activity assays

Cell-surface protein analysis was performed using two methods: cell-surface biotinylation and flow cytometry. To biotinylate the cell-surface proteins, the Pierce™ Cell Surface Protein Isolation Kit (Thermo Scientific) was utilized following the manufacturer's instructions. Briefly, exposed primary amines of proteins on the surface of proliferating and senescent hVSMCs were labeled with a cleavable biotinylation reagent. Cells were harvested and lysed, surface proteins purified with an avidin resin, and proteins assessed by western blot analysis. To analyze cells by flow cytometry, proliferating and senescent hVSMCs were washed with phosphate-buffered saline (PBS), trypsinized and pelleted. Cells were resuspended in 100 μL of 1x PBS, incubated with 5 μL of human TruStain FcX™ Fc blocking reagent for 5 min, and incubated for 15 min with 5 μL anti-human CD26-APC (BD Biosciences, Clone M-A261) at 25°C in the dark. Cells were then washed twice with 1x PBS, resuspended in 200 μL of 1x PBS, and stained with 2 μg/mL of propidium iodide (PI) for 5 min to assess cell viability. Fluorescence intensity was analyzed using BD FACS Canto™ II. A DPP4 Activity Assay Kit (Abcam) was used to determine DPP4 proteolytic activity in hVSMCs, monkey, and mouse serum, following the manufacturer's instructions.

Atherosclerotic lesion analysis – additional details

Lesion size in the aortic arch was determined by quantitative morphometry using the ImageJ program. Aortic root was frozen in OCT medium and sectioned. Four transverse serial sections spaced 70-100 μm apart from the aortic sinus to disappearance of valve cusps per aortic root were stained with Oil Red O; positive stained areas quantitated as % total area by quantitative morphometry.

Single-cell preparation, and single-cell RNA-seq and CITE-seq analysis – additional details

For the single-cell libraries, between 5,000 to 10,000 single cells were used for GEM generation. The cDNAs were then prepared and separated by SPRI beads for cDNA library and protein library and checked on the Agilent Bioanalyzer with High Sensitivity DNA kit (Agilent). Prepared cDNAs were used for library preparation for sequencing and final libraries were checked on the Agilent Bioanalyzer with DNA 1000 kit (Agilent).

After acquiring read count matrices and subsequently analyzed in R using the Seurat package, standard quality control filtering was applied to each sample to eliminate low-quality cells and potential doublets from downstream analysis. Filtering also removed cells containing more than 25% mitochondrial genes, expressing fewer than 200 or greater than 6,000 transcripts, and more than 2,000 ADT counts from the final analysis. Genes that were detected in less than 3 cells were excluded from the analysis. For each sample, ADT counts were normalized using the "CLR" method, and RNA data with "LogNormalize" method followed by running the FindVariableFeatures function to

select 2,000 most variable genes for dimensionality reduction. The FindIntegrationAnchors function was applied to choose anchors for data integration. After performing Principal Component Analysis (PCA), the top 40 PCA dimensions were determined by the ElbowPlot method and used to create the Uniform Manifold Approximation and Projection (UMAP) with the resolution parameter set to 0.2. Differentially expressed marker genes for each cluster were identified with the FindAllMarkers function, and the FindMarkers function was used to find differentially expressed genes between experimental conditions. The main cell types were identified using marker genes of each cluster in combination with data from literature. For clustering of SMC subpopulations, the analysis was rerun on the SMC subset and UMAP was created using 30 PCs and resolution 0.2.

HVSMC mass spectrometry and proteomics – additional details

Briefly, the pellet was resuspended in 5% SDS followed by reduction (5 mM DTT), alkylation (20 mM iodoacetamide) and loading on S-trap (ProtiFi)-based digestion using trypsin. Before mass spectrometry analysis, samples were desalted using a 96-well plate filter (Orochem) packed with 1 mg of Oasis HLB C-18 resin (Waters). Samples were loaded onto a Dionex RSLC Ultimate 300 (Thermo Scientific), coupled online with an Orbitrap Fusion Lumos (Thermo Scientific). Chromatographic separation was performed with a two-column system, consisting of a C-18 trap cartridge (300 µm ID, 5 mm length) and a picofrit analytical column (75 µm ID, 25 cm length) packed in-house with reversed-phase Repro-Sil Pur C18-AQ 3 µm resin. To analyze the proteome, peptides were separated using a 180 min gradient from 4-30% buffer B (buffer A: 0.1% formic acid, buffer B: 80% acetonitrile + 0.1% formic acid) at a flow rate of 300 nl/min. The mass spectrometer was set to acquire spectra in a data-dependent acquisition (DDA) mode. Proteome raw files were searched using Proteome Discoverer software (v2.4, Thermo Scientific) using SEQUEST search engine. The search for total proteome included variable modification of N-terminal acetylation, and fixed modification of carbamidomethyl cysteine. Trypsin was specified as the digestive enzyme with up to 2 missed cleavages allowed. Mass tolerance was set to 10 pm for precursor ions and 0.2 Da for product ions. Peptide and protein false discovery rate was set to 1%.

Immunofluorescence and staining

Immunofluorescence analysis to detect FX (antibody) and p21 (antibody) in aortic roots was performed commercially by iHisto (iHisto.io). Oil Red O and Hematoxylin and Eosin (H&E) staining of aortic roots was performed by iHisto.

Bioplex analysis

For multiplex analysis, serum was thawed and centrifuged at 16,000 x g for 4 min. Custom human or murine Luminex Assay kits were designed by R&D Biosystems to include the following analytes: DPP4, GDF-15, TIMP1, SERPINE1, C2, C5A, C9, VEGFC, Coagulation Factor III, Coagulation Factor XIV, MMP1, MMP12, CXCL1, CCL3, and VEGF. Serum was diluted 1:10 using the Calibrator Diluent RD6-52 provided in the kit. Standards (provided with the kit), Blanks, and Serum were incubated with the microparticle cocktail for 2 h at 25°C, followed by incubation with Biotin-Antibody cocktail for 1 h. The final incubation lasted 30 min with Streptavidin-PE and shaking at 25°C prior to running the plate on the Bio-Rad Bioplex-200 Instrument. Each incubation was followed by washing 3 times with Wash Buffer (provided in the kit). Instrument settings were adjusted to the following: 50 µl sample volume, Bio-Plex MagPlex Beads (Magnetic), Double Discriminator Gates set at 8,000 and 23,000, low RP1 target value for the CAL2 setting, 50 count/region. The results were analyzed with the Bio-Plex Manager software.

Morphometric analysis of DPP4 in human abdominal advanced atherosclerotic plaque with lipid necrotic core/focal necrosis

Plaque fibrous cap, plaque shoulder, and plaque base of each atherosclerotic plaque were evaluated for DPP4 density (brown color); 3 images taken at x40 magnification were used to identify the proportion of stained area relative to the corresponding total area using the DAB immunostaining. For plaque core, individual DPP4 densities (brown color) were measured in the lipid necrotic core area, including its enclosed boundary of each atherosclerotic plaques with 16-60 images taken at 10x and x40 magnification was expressed as the proportion of stained area to the corresponding total area, using DAB immunostaining.

Human tissue mass spectrometry – additional details

Briefly, 10 frozen aortic tissue samples were processed for the study. Tissues were lysed in lysis buffer containing 2% SDS and sonicated using Sonic Dismembrator. Samples were centrifuged at 13,000 rpm for 12 min. Protein concentrations were measured using the BCA method and 100 µg of protein from each of the 10 samples was processed for trypsin digestion, followed by TMT-multiplex labeling using two TMT-11plex sets. Unique TMT tags from each set were used to label 75 µg of trypsin-digested peptides from each of the 20 digests; one TMT tag was used to label a master mixture. The LC-MS/MS analysis was carried out using a Thermo Scientific Orbitrap Exploris 240 Mass Spectrometer and a Thermo Dionex UltiMate 3000 RSLCnano System.

Analysis. TMT-tag based quantification was used to determine the relative abundance of proteins identified from 24 fractions for each TMT set. Relative protein abundance was calculated using the ratio of abundance determined by the TMT-tags. A normalization of the ratio (relative abundance) was performed using the summed reporter ion intensities.

Rhesus monkey study

Samples were contributed from a previous study in which young (6-15 years-old, n = 4) and old male rhesus *(Macaca mulatta)* monkeys (16-28 years-old, n = 4), were fed a diet of high fat and cholesterol (HFD) for two years. Young and old control samples were collected from monkeys (n = 4 each) on an unrelated study that were maintained on a ND.

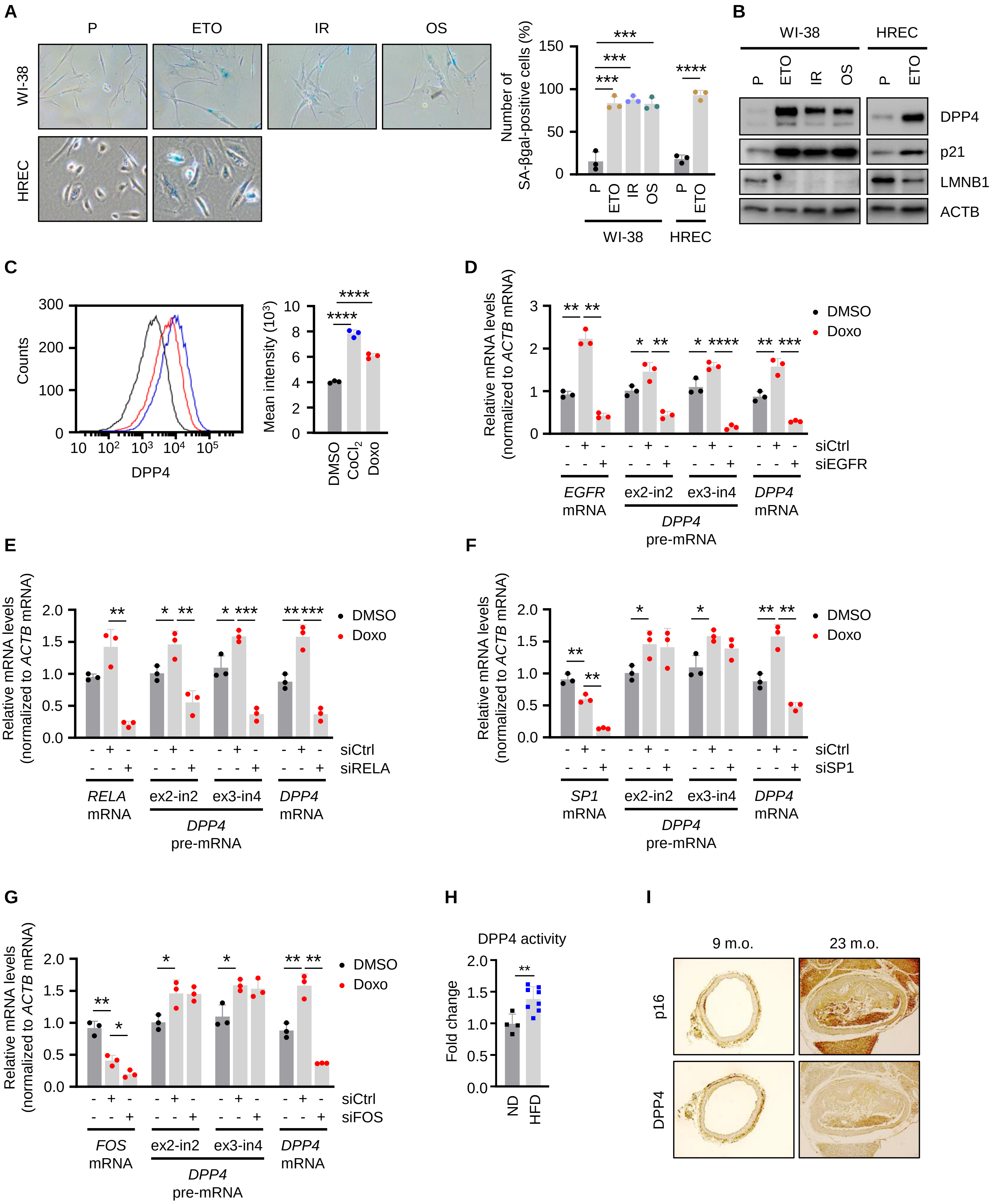

Figure S1. DPP4 levels in senescent cell culture models and animal models of vascular disease and aging.
(A) SA-βgal staining of WI-38 and HREC proliferating (P) cells and cells rendered senescent by exposure to etoposide (ETO), ionizing radiation (IR), or oxidative stress (OS) (*left*), and quantified as percentage SA-βgal-positive relative to proliferating cells (*right*) (20x). **(B)** WI-38 cells and HRECs were rendered senescent as described in (A) and western blot analysis was used to assess the levels of DPP4, p21, LMNB1, and loading control ACTB. **(C)** *Left*, flow cytometry analysis of cell-surface expression of DPP4 in hVSMCs treated with DMSO, $CoCl_2$, or Doxo. Right, quantification of the mean intensity of the signals for DPP4 in each of the described conditions. **(D-G)** RT-qPCR analysis of transcription factor silencing and DPP4 pre-mRNA and mature mRNA following DMSO or Doxo treatment. Silencing of *EGFR* mRNA **(D)**, *RELA* mRNA **(E)**, *SP1* mRNA **(F)**, and *FOS* mRNA **(G)** was assessed by RT-qPCR analysis. **(H)** *Ldlr-/-* mice were fed control or high-fat diets (HFD) for 16 weeks and DPP4 activity was measured from serum. **(I)** Aortas from *ApoE*$^{-/-}$ mice that were 9 or 23 months old (m.o.) were analyzed for p16 and DPP4 (50x). Immunohistochemistry (IHC), signals were displayed as brown staining. Significance was established using Student's t-test. *, p≤0.05; **, p≤0.01; ***, p≤0.001.

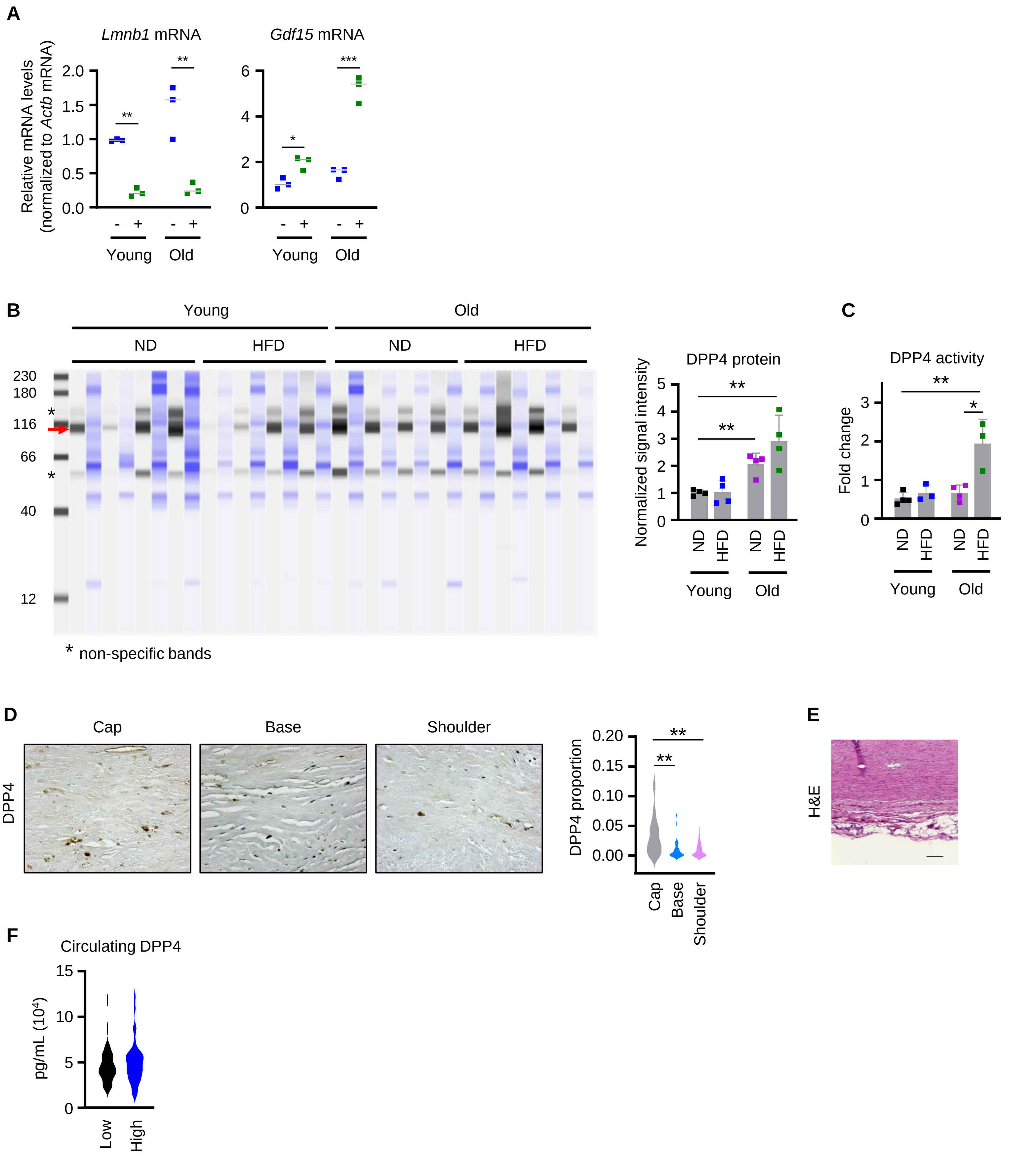

Figure S2. DPP4 expression in young and old vascular cells and models of vascular disease and aging.
(A) RT-qPCR analysis of *Lmnb1* and *Gdf15* mRNAs normalized to *Actb* mRNA from DPP4-positive and negative mouse VSMCs from young and old mice. **(B)** *Left*, Jess Western Blot system visualization with protein normalization (blue bands) for aortic tissues from monkeys that were young and ND, young and fed HFD, old and fed ND, and old and fed HFD (black bands); n = 3-4 per group. The red arrow indicates bands at the molecular weight of DPP4, asterisks indicate non-specific bands. The first column is a molecular weight marker. *Right*, quantification of Jess western blot gel image. **(C)** DPP4 activity levels in young and old rhesus monkeys fed a ND or HFD assessed by DPP4 activity assay kit. **(D)** *Left,* immunohistochemical analysis of DPP4 in the cap, base, and shoulder area of a human atherosclerotic plaque. *Right*, quantification of (D) as a proportion of the entire tissue area (100x). **(E)** H&E staining of a representative region of atherosclerotic tissue from an older person (10x). **(F)** Human circulating DPP4 levels were measured in serum samples from people with low and high Framingham risk score (FRS) designations (n=50 per group). Data represent the mean values ±SD from three biological replicates, unless otherwise indicated. Significance was established using Student's t-test. *, p≤0.05; **, p≤0.01; ***, p≤0.001.

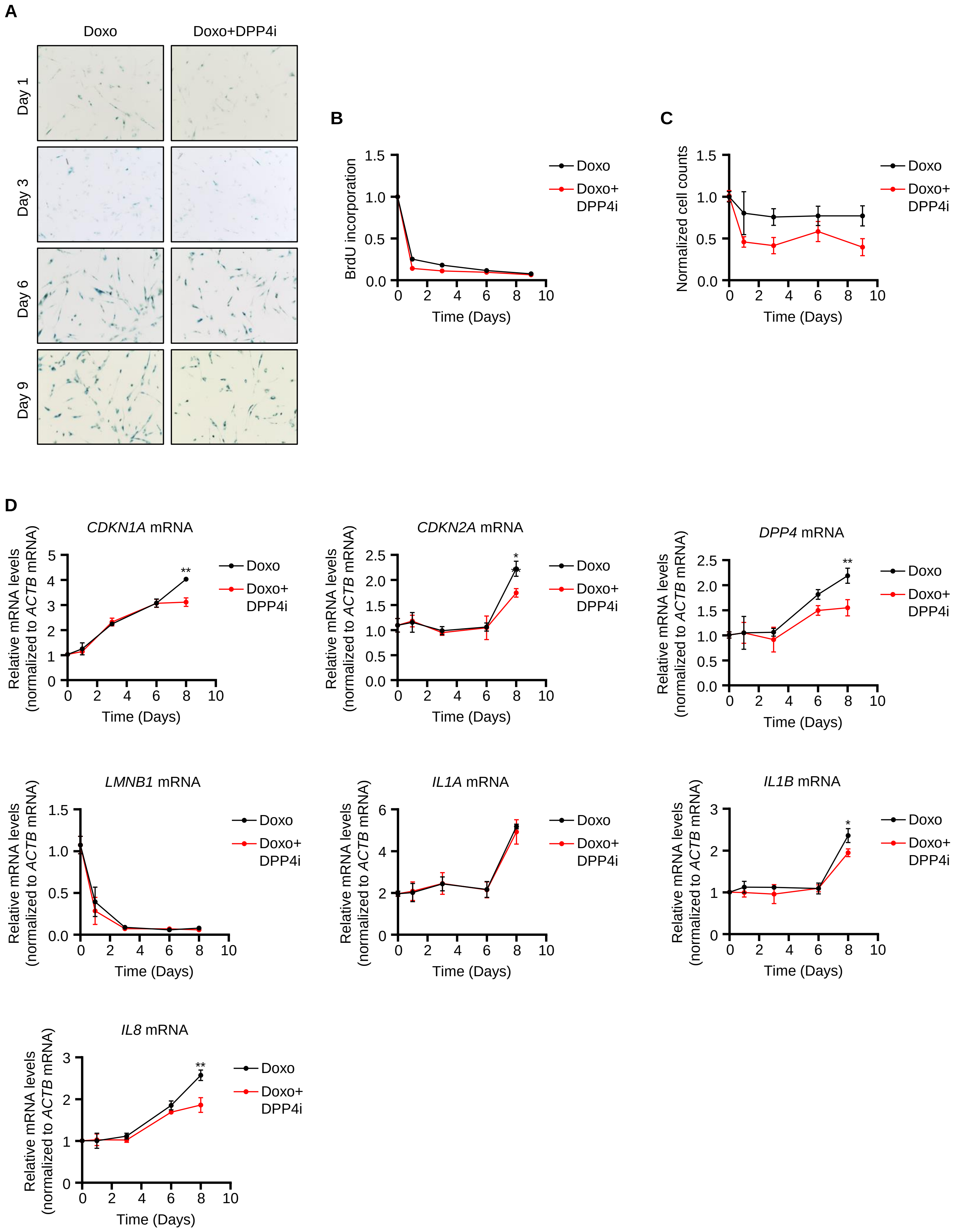

Figure S3. DPP4 inhibitor time course in senescent hVSMCs.
(A) SA-βgal staining (20x), **(B)** BrdU incorporation, and **(C)** normalized cell counts as a surrogate for viability was measured in hVSMCs treated with Doxo or Doxo + DPP4i for the indicated times. **(D)** RT-qPCR analysis of *CDKN1A, CDKN2A, DPP4, LMNB1, IL1A, IL1B,* and *IL8* mRNAs normalized to *ACTB* mRNA in hVSMCs treated with Doxo or Doxo + DPP4i for the indicate times. Significance was established using Student's t-test. *, p≤0.05; **, p≤0.01; ***, p≤0.001.

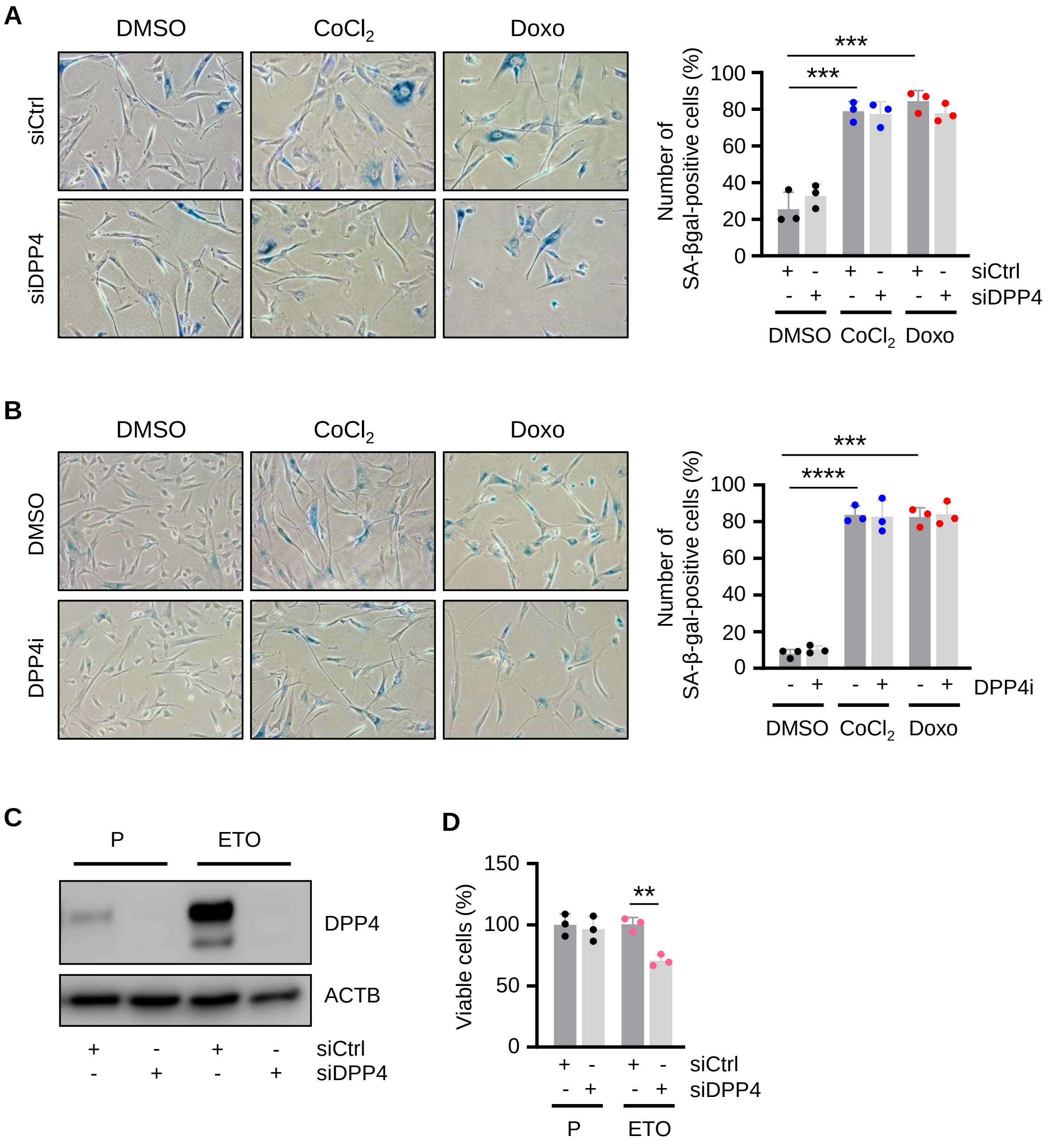

Figure S4. DPP4 silencing or inhibition in senescent cells.
(A) HVSMCs transfected with siCtrl or siDPP4 and treated with DMSO, $CoCl_2$, or Doxo for 7 days were stained for SA-βgal activity (*left*) and quantified as the percentage of SA-βgal-positive cells (*right*) (20x). **(B)** *Left*, hVSMCs simultaneously treated with DMSO or DPP4i along with DMSO, $CoCl_2$, or Doxo for 7 days were stained for SA-βgal activity and quantified as the percentage of SA-βgal-positive cells (*right*) (20x). **(C)** Western blot analysis of DPP4 expression in proliferating (P) and senescent (induced by ETO) WI-38 fibroblasts. ACTB protein levels were visualized as a loading control. **(D)** Percentage of viable proliferating (P) or ETO-induced senescent WI-38 cells transfected with siCtrl or siDPP4. Data represent the mean values ±SD from three biological replicates, unless otherwise indicated. Significance was established using Student's t-test. *, $p \leq 0.05$; **, $p \leq 0.01$; ***, $p \leq 0.001$.

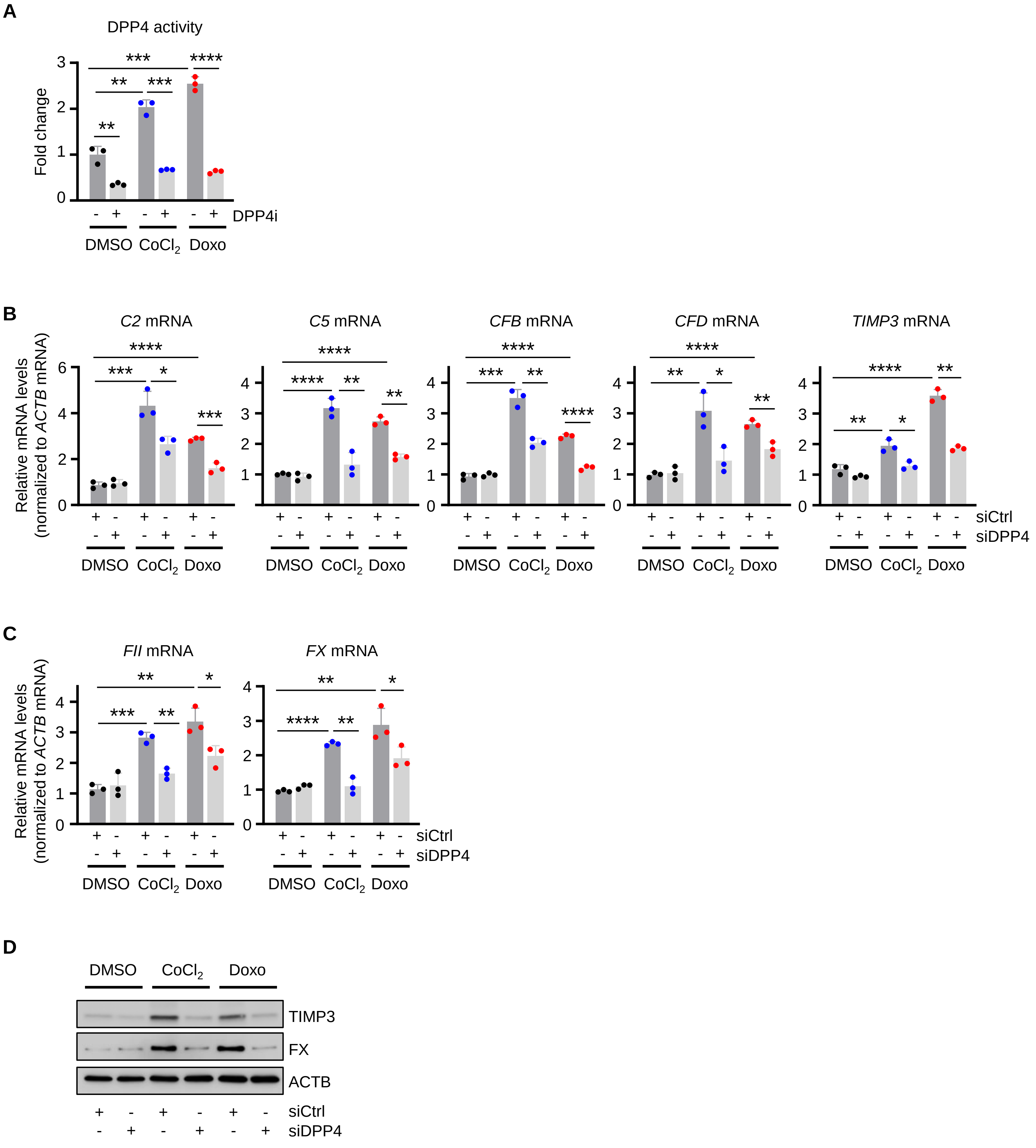

Figure S5. Validation of targets identified after inhibiting or silencing DPP4.
(A) DPP4 activity measured in lysates treated with DPP4i for Mass Spectrometry in Figure 3A. **(B, C)** RT-qPCR analysis of the levels of *C2*, *C5*, *CFB*, *CFD*, *TIMP3, FII*, and *FX* mRNAs normalized to *ACTB* mRNA in hVSMCs transfected with siCtrl or siDPP4 and treated with DMSO, $CoCl_2$, or Doxo for 7 days. **(D)** Western blot analysis of proteins TIMP3, FX, and normalization control ACTB, in hVSMCs transfected with siCtrl or siDPP4 and treated with DMSO, $CoCl_2$, or Doxo for 7 days. Data represent the mean values ±SD from three biological replicates, unless otherwise indicated. Significance was established using Student's t-test. *, p≤0.05; **, p≤0.01; ***, p≤0.001.

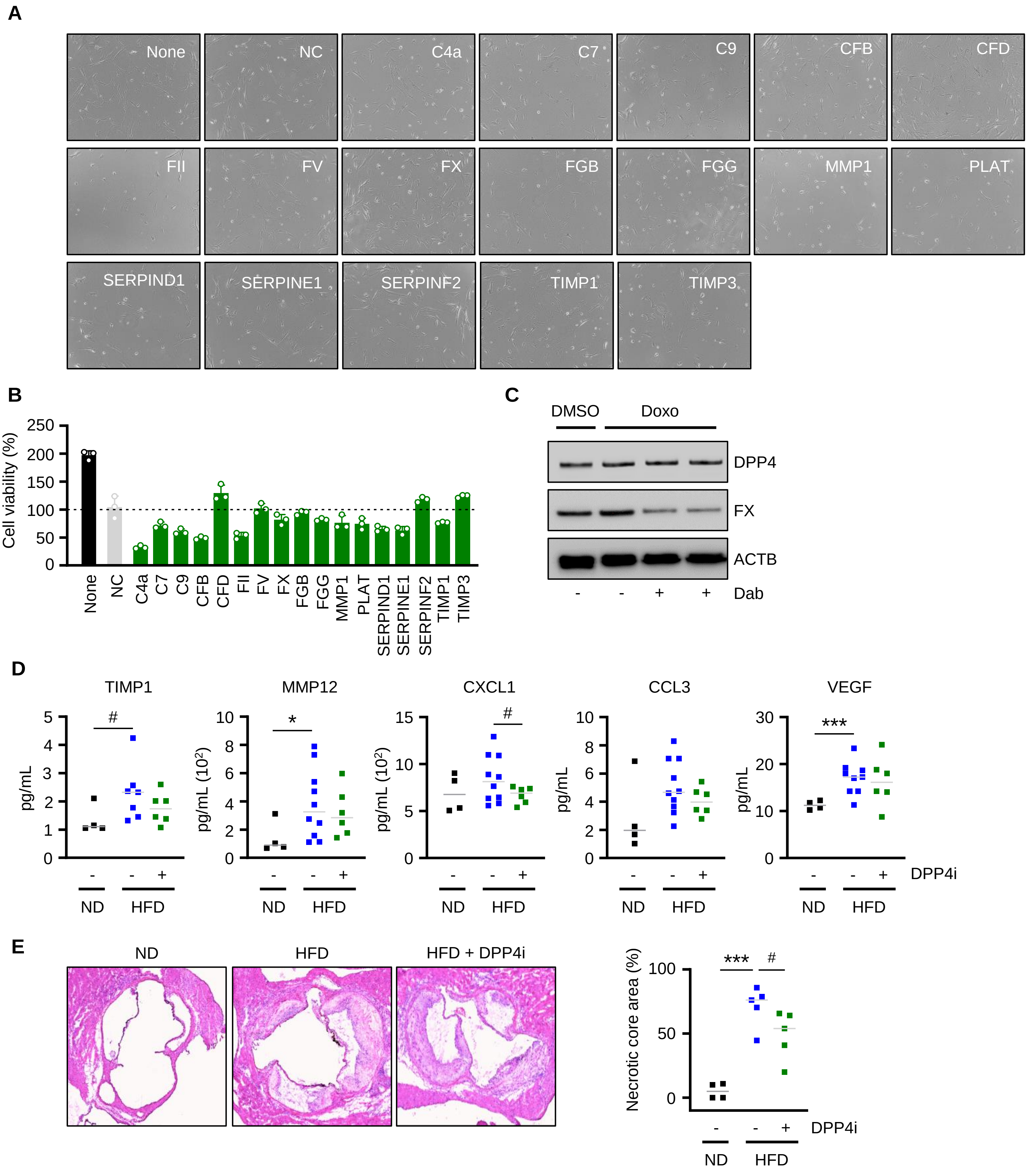

Figure S6. siRNA screening and drug treatments in senescent hVSMCs.
(A) Brightfield images of hVSMCs transfected with the indicated siRNAs and treated with Doxo for 48 h (20x). **(B)** Percentages of cell viability following transfection with the indicated siRNAs and treated with Doxo for 72 h. **(C)** Western blot analysis of DPP4, FX, and loading control ACTB in hVSMCs treated with DMSO or Doxo +/- Dab. **(D)** Bioplex analysis of circulating TIMP1, MMP12, CXCL1, CCL3, and VEGF in serum from *Ldlr*$^{-/-}$ mice fed a regular diet, HFD, or HFD + DPP4i. **(E)** Representative images of aortic roots from mice in ND, HFD, and HFD + DPP4i treatment groups, stained with H&E (*left*) percentage of necrotic core area (*right*) (100x). Data represent the mean values ±SD from three biological replicates, unless otherwise indicated. Significance was established using Student's t-test or one-way ANOVA. #=0.07; *, p≤0.05; **, p≤0.01; ***, p≤0.001.

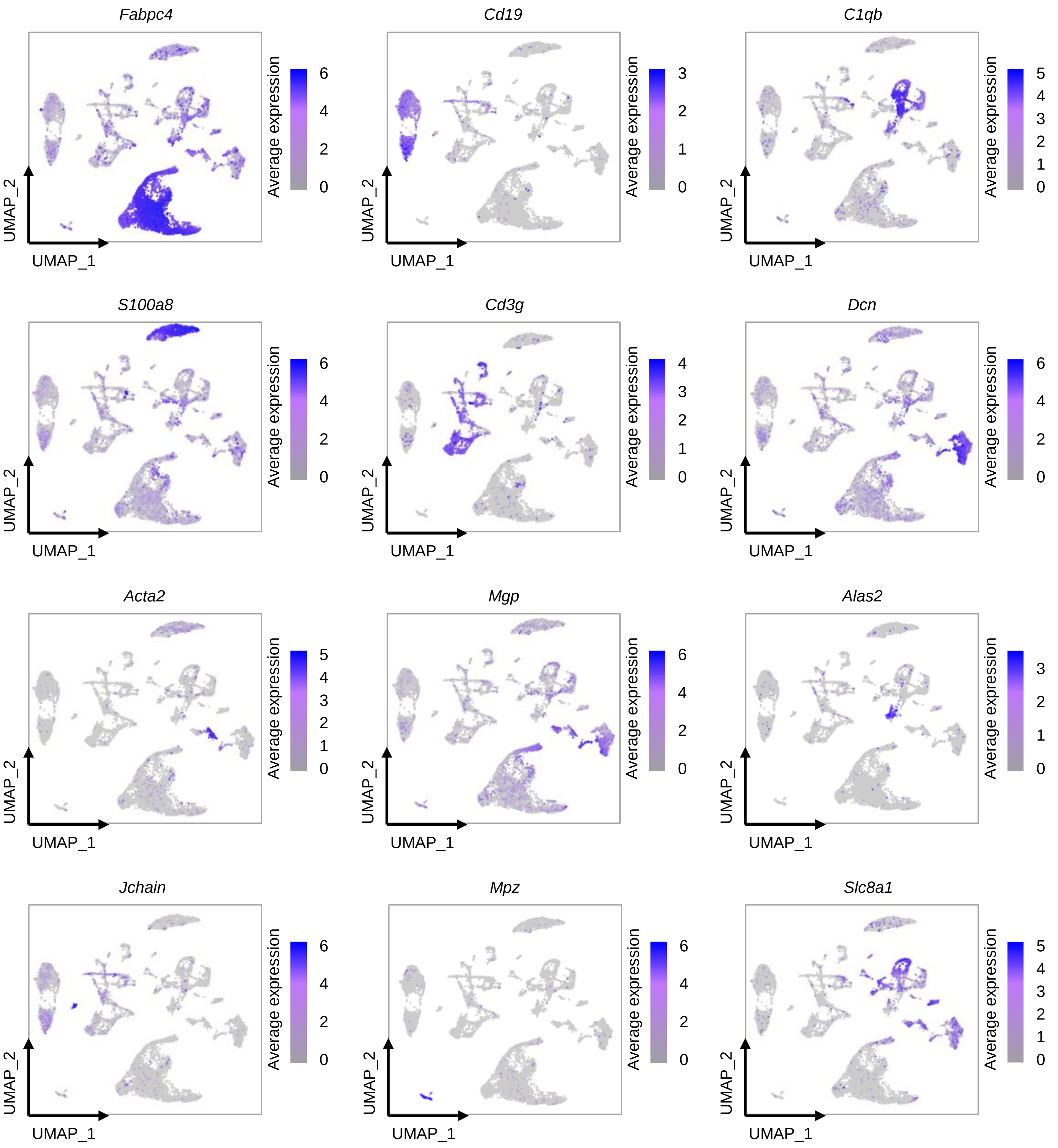

Figure S7. Aortic single-cell RNA-seq analysis cluster UMAPs from *Ldlr*$^{-/-}$ mice.
UMAPs of unsupervised clustering of each cluster with a marker gene representing the cell type.

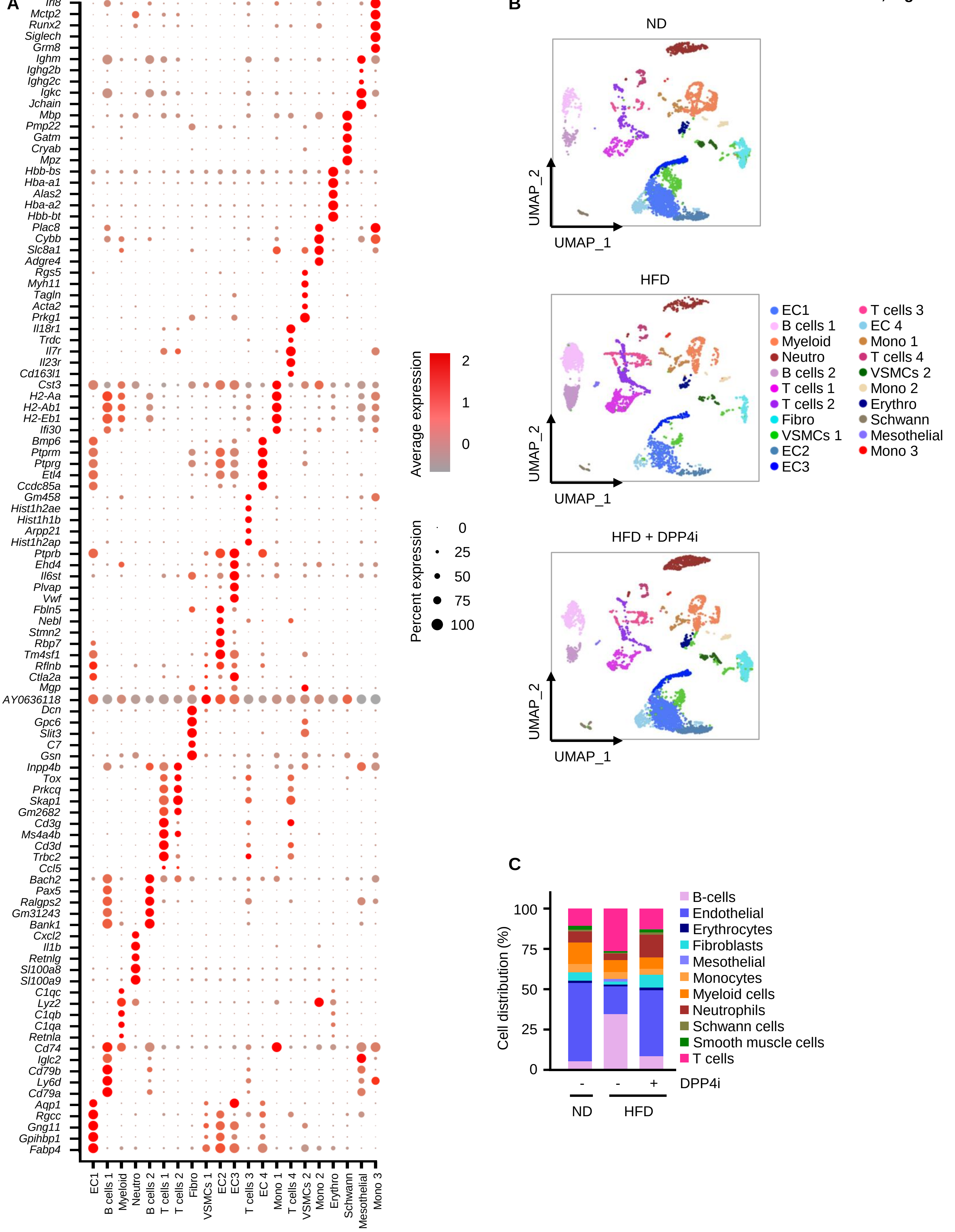

Figure S8. Single-cell RNA-seq analysis and identification of DPP4-expressing cells by CITE-seq analysis in *Ldlr*$^{-/-}$ atherosclerosis mouse model.
(A) Top 5 genes with highest average expression in each cell cluster compared to all other clusters. The intensity of color indicates gene average expression and dot size represents the proportion of cells expressing a specific gene. **(B)** UMAP of unsupervised clustering of all aortic cells. **(C)** Aortic cell distribution in ND, HFD, and HFD + DPP4i represented as percentage of total cells.

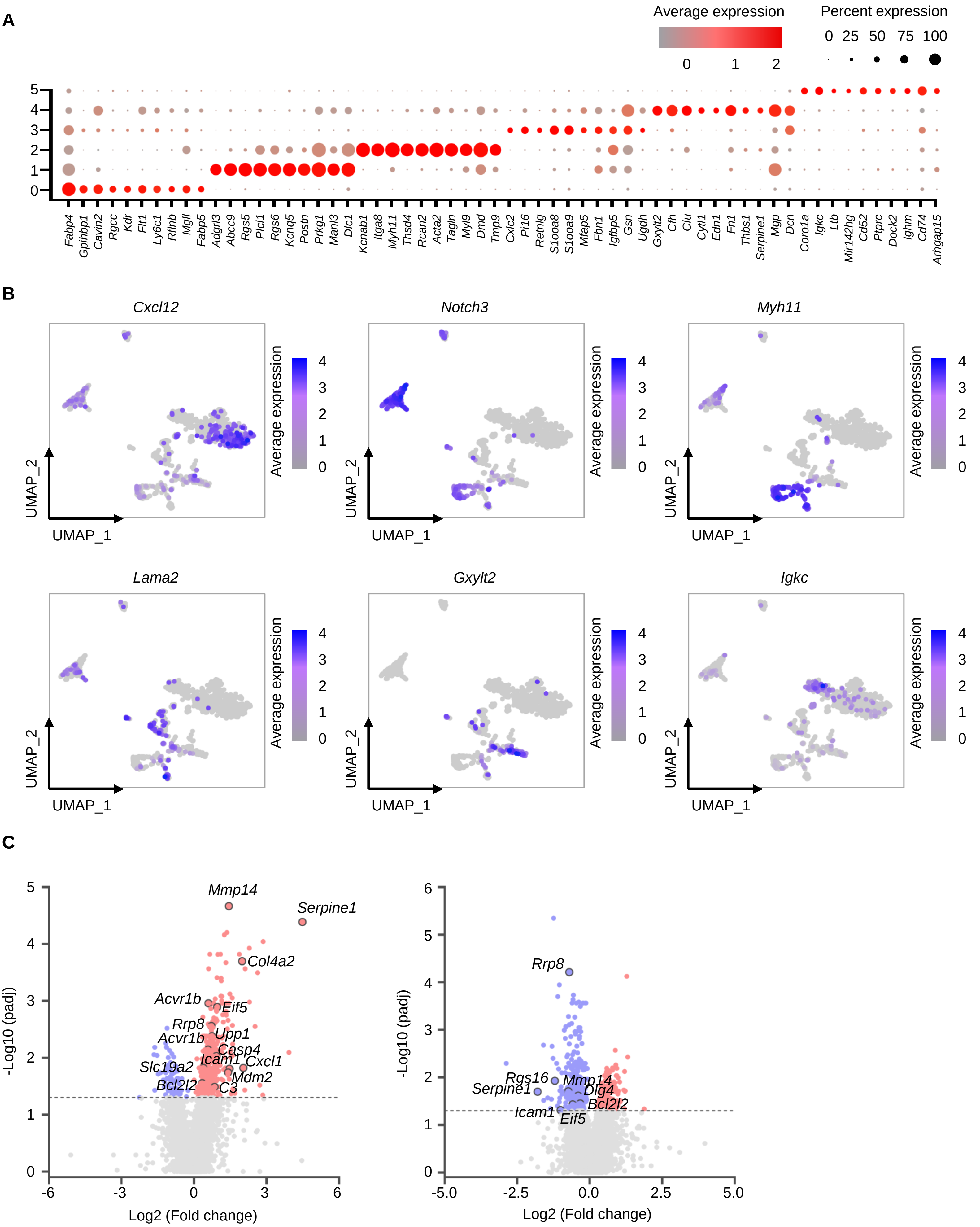

Figure S9. VSMC subset analysis.
(A) Top 10 genes with highest average expression for each subpopulation of VSMCs. **(B)** UMAPs of unsupervised clustering of each VSMC subpopulation with a representative marker gene. **(C)** Volcano plots of differentially expressed genes in cluster 4: HFD compared to ND (*left*) and HFD + DPP4i compared to HFD (*right*).
