# Supplementary material for: DPP4 inhibition impairs senohemostasis to improve plaque stability in atherosclerotic mice
Source: J Clin Invest. 2023 Jun 15;133(12):e165933. doi: 10.1172/JCI165933 (PMC10266795; doi:10.1172/JCI165933)
Supplement: Supplemental table 2 [file jci-133-165933-s217.pdf]

Supplemental Table 2

RT-qPCR Primers	
Human	
CFD_Forward	GACACCATCGACCACGAC
CFD_Reverse	GTTGACTATGCCCCAGCC
SERPINE1_Forward	GTGGACTTTTCAGAGGTGGAG
SERPINE1_Reverse	GAAGTAGAGGGCATTCACCAG
FII_Forward	ATGTCTGGAAGGTAACTGTGC
FII_Reverse	CAGGATGGGTAGTGGAGTTG
TIMP3_Forward	TGATGGCAAGATGTACACGG
TIMP3_Reverse	GAAGTCACAAAGCAAGGCAG
CFB_Forward	ACCAAAAGACTGTCAGGAAGG
CFB_Reverse	GAGAGTGTAACCGTCATAGCAG
FX_Forward	TCTTTGAGGACAGCGACAAG
FX_Reverse	ATCCTTCTAAACAGGTGCAGG
C2_Forward	CTTGAATGGGAGCAAACTGAAC
C2_Reverse	GCATAGGAACTGGTCTGTCAC
CDKN1A_Forward	AGTCAGTTCCTTGTGGAGCC
CDKN1A_Reverse	CATGGGTTCTGACGGACAT
BCL2L2_Forward	GATGGTGGCCTACCTGGAGA
BCL2L2_Reverse	AGAGCTGTGAACTCCGCCCA
BCL2L1_Forward	GAGCTGGTGGTTGACTTTCTC
BCL2L1_Reverse	TCCATCTCCGATTCAGTCCCT
DPP4_Forward	CAAATTGAAGCAGCCAGACA
DPP4_Reverse	CACACTTGAACACGCCACTT
DPP4 (in.2-ex.2)_Forward	GCGCTTGTCACCATCATCACC
DPP4 (in.2-ex.2)_Reverse	GGTCCAACCCCACGCAAAA
DPP4 (in.3-ex.4)_Forward	TTTGATCTGGGTGTTTTCTCTCTT
DPP4 (in.3-ex.4)_Reverse	TGAGCTGTTTCCATATTCAGCA
LMNB1_Forward	CTCAAGGCGCTCTACGAGAC
LMNB1_Reverse	GCGAAACTCCAACTCCTCAG
C5_Forward	TCACTGGAGACTTGGTTTGG
C5_Reverse	ACTCCTTTCGTCTGCTAATGG
RelA_Forward	ATGTGGAGATCATTGAGCAGC
RelA_Reverse	CCTGGTCCTGTGTAGCCATT
EGFR_Forward	CAGACCGGACGACAGGC
EGFR_Reverse	ATACTGGACGGAGTCAGGGG
Fos_Forward	TACTACCACTCACCCGCAGA
Fos_Reverse	CGTGGGAATGAAGTTGGCAC
SP1_Forward	CATCCCCTTTGGCTCTGCTG
SP1_Reverse	TGGCACCCTGTGAAAGTTGT
ACTB_Forward	GCACAGAGCCTCGCCTT
ACTB_Reverse	GTTGTCGACGACGAGCG
Mouse	
mLmnb1_Forward	GGGAAGTTTATTCGCTTGAAGA
mLmnb1_Reverse	ATCTCCCAGCCTCCCATT
mCdkn1a_Forward	TTGCCAGCAGAATAAAAGGTG
mCdkn1a_Reverse	TTTGCTCCTGTGCGGAAC
mActb_Forward	TTCTTTGCAGCTCCTTCGTT
mActb_Reverse	ATGGAGGGGAATACAGCCC
mIl1a_Forward	AGCGCTCAAGGAGAAGACC
mIl1a_Reverse	CCAGAAGAAAATGAGGTCGG
mGdf15_Forward	CTGGCAATGCCTGAACAACG
mGdf15_Reverse	GGTCGGGACTTGGTTCTGAG
mIl1b_Forward	GAAATGCCACCTTTTGACAGTG
mIl1b_Reverse	TGGATGCTCTCATCAGGACAG
