# Supplementary material for: DPP4 inhibition impairs senohemostasis to improve plaque stability in atherosclerotic mice
Source: J Clin Invest. 2023 Jun 15;133(12):e165933. doi: 10.1172/JCI165933 (PMC10266795; doi:10.1172/JCI165933)
Supplement: Supplemental table 5 [file jci-133-165933-s220.pdf]

Supplemental Table 5. Key resources table

REAGENT or RESOURCE	SOURCE	IDENTIFIER
Antibodies		
P53	Cell Signaling Technology	18032S
P21	Cell Signaling Technology	2947S
Beta Actin	Santa Cruz Biotechnology	sc-47778
Lamin B1	Abcam	ab16048
BCL2L2	Cell Signaling Technology	2724
BCL2L1	Cell Signaling Technology	2764
TIMP3	Abcam	ab39184
FX	Abcam	ab196023
DPP4	Cell Signaling Technology	67138
P16	Abcam	ab54210
VDAC1	Santa Cruz Biotechnology	sc-58649
DPP4 (immunohistochemistry)	Abcam	ab225901
Cdkn2a (immunohistochemistry)	Abcam	ab54210
aSMA	Cell Signaling Technology	19245
EGFR	Cell Signaling Technology	2232S
NF-κB p65	Cell Signaling Technology	8242
APC anti-human CD26 Antibody	BD Biosciences	563670
APC anti-mouse CD26 Antibody	BioLegend	137807
TotalSeq™-C0987 anti-Allophycocyanin (APC) Antibody	BioLegend	408007
FITC anti-mouse CD31 Antibody	BioLegend	102405
PE/Cyanine 7 anti-mouse CD45 Antibody	BioLegend	103113
Pacific Blue anti-mouse TER-119 Antibody	BioLegend	116232
Chemicals, peptides, and recombinant proteins		
Lipofectamine RNAiMax	Thermo Fisher Scientific	13778150
RNase OUT	Invitrogen	10777019
Vildagliptin	Selleckchem	S3033
Dabigatran	Selleckchem	S2196
ZVAD-FMK	Selleckchem	S7023
$CoCl_2$	Sigma-Aldrich	C8661
Doxorubicin	Sellechkchem	E2516
TriPure Isolation Reagent	Roche	11667165001
TRIzol™ LS Reagent	Thermo Fisher Scientific	10296028
Lipofectamine RNAiMax	Thermo Fisher Scientific	13778150
Etoposide	Selleckchem	S1225
Human TruStain FcX™ Fc Blocking reagent	BioLegend	422301
Mouse TruStain FcX™ Fc Blocking reagent	BioLegend	101320
Collagenase II	Sigma-Aldrich	9001-12-1
Elastase	Worthington	LS 002292
DMEM	Gibco	11965092
FBS	Gibco	26140079
Penn/Strep	Gibco	15140122
Sodium Pyruvate	Gibco	11360070
Non-essential amino acids	Gibco	11140050
VascuLife® SMC Medium Complete Kit	LifeLine	LL-0014

Propidium Iodide	Invitrogen	P1304MP
Trypsin-EDTA (0.05%), phenol red	Gibco	25300054
Critical commercial assays		
DPP4 Activity Assay	Abcam	ab204722
Caspase-Glo 3/7 Assay	Promega	G8091
Senescence-β-Galactosidase Staining Kit	Cell Signaling Technology	9860S
Direct-zol RNA Miniprep	Zymo Research	R2051
Maxima First Strand cDNA Synthesis Kit for RT-qPCR	Thermo Fisher Scientific	K1641
Pierce™ Cell Surface Protein Isolation Kit	Thermo Fisher Scientific	89881
Chromium Next GEM Single Cell 3' Kit v3.1	10X Genomics	1000268
Chromium Next GEM Chip G Single Cell Kit	10X Genomics	1000120
Bioanalyzer DNA 1000 kit	Agilent	5067-1504
High Sensitivity DNA kit	Agilent	5067-4626
Deposited data		
Raw and analyzed data	This paper	
Experimental models: Cell lines		
WI-38 Fibroblasts	Coriell Institute for Medical Research	AG07217
Human Coronary Artery Smooth Muscle Cells (hVSMCs)	LifeLine Cell Technology	FC-0031
Human renal mixed epithelial cells (HRECs)	ATCC	PCS-400-012
Experimental models: Organisms/strains		
LDLR-/- mouse strain (B6.129S7-Ldlrtm1Her/J)	The Jackson Laboratory	002207
C57Bl/6 mouse strain	Charles River Laboratory	
Oligonucleotides		
siDPP4	Dharmacon	L-004181-00-0005
siCtrl	Dharmacon	D-001810-10-05
siRNA screening plate	Dharmacon	Table S1
RT-qPCR primers	IDT	Table S2
siSp1	Dharmacon	L-026959-00-0005
siEGFR	Dharmacon	L-003114-00-0005
siFos	Dharmacon	L-003265-00-0005
Software and algorithms		
Seurat package, version 4.1.0	Hao et al., 2021	
Other		
Harlan atherogenic diet	Envigo	TD.88137
